# Simulation-based model checking approach to cell fate specification during *Caenorhabditis elegans *vulval development by hybrid functional Petri net with extension

**DOI:** 10.1186/1752-0509-3-42

**Published:** 2009-04-27

**Authors:** Chen Li, Masao Nagasaki, Kazuko Ueno, Satoru Miyano

**Affiliations:** 1 Human Genome Center, Institute of Medical Science, University of Tokyo, 4-6-1 Shirokanedai, Minato-ku, Tokyo 108-8639, Japan

## Abstract

**Background:**

Model checking approaches were applied to biological pathway validations around 2003. Recently, Fisher *et al*. have proved the importance of model checking approach by inferring new regulation of signaling crosstalk in *C. elegans *and confirming the regulation with biological experiments. They took a discrete and state-based approach to explore all possible states of the system underlying vulval precursor cell (VPC) fate specification for desired properties. However, since both discrete and continuous features appear to be an indispensable part of biological processes, it is more appropriate to use quantitative models to capture the dynamics of biological systems. Our key motivation of this paper is to establish a quantitative methodology to model and analyze *in silico *models incorporating the use of model checking approach.

**Results:**

A novel method of modeling and simulating biological systems with the use of model checking approach is proposed based on hybrid functional Petri net with extension (HFPNe) as the framework dealing with both discrete and continuous events. Firstly, we construct a quantitative VPC fate model with 1761 components by using HFPNe. Secondly, we employ two major biological fate determination rules – Rule I and Rule II – to VPC fate model. We then conduct 10,000 simulations for each of 48 sets of different genotypes, investigate variations of cell fate patterns under each genotype, and validate the two rules by comparing three simulation targets consisting of fate patterns obtained from *in silico *and *in vivo *experiments. In particular, an evaluation was successfully done by using our VPC fate model to investigate one target derived from biological experiments involving hybrid lineage observations. However, the understandings of hybrid lineages are hard to make on a discrete model because the hybrid lineage occurs when the system comes close to certain thresholds as discussed by Sternberg and Horvitz in 1986. Our simulation results suggest that: Rule I that cannot be applied with qualitative based model checking, is more reasonable than Rule II owing to the high coverage of predicted fate patterns (except for the genotype of *lin-15ko; lin-12ko *double mutants). More insights are also suggested.

**Conclusion:**

The quantitative simulation-based model checking approach is a useful means to provide us valuable biological insights and better understandings of biological systems and observation data that may be hard to capture with the qualitative one.

## Background

Model checking is a successful method for automatic verification of software and reactive systems [1], which is usually applied to ensure consistency and correctness of designed models. Practiced verification methods in most cases are still simple simulation and testing. While simple simulation and testing provide a part of the possible results of a model, model checking can conduct an exhaustive exploration of all possible behaviors [1-4].

Among the features, one merit of using model checking is that it is possible to verify a set of rules defined by users. Recently, the size of targeting network becomes enlarged and difficult to check all rules and their combinations by each user, especially, biologist. To solve this, around 2003, several attempts were launched to apply model checking approaches to biological pathway validations [5-12]. With the aid of model checking approach, one can obtain answers to questions such as "what is the probability that the gene finally expressed?" and "does this reaction always lead to DNA fragmentation?" In 2007, Fisher *et al*. proved the importance of model checking approach by inferring the new regulation of inductive and lateral signaling crosstalk of *C. elegans *and confirming the regulation with biological experiments [9]. The approach was applied on a discrete model by using one of the model checking languages named *reactive modules *[13]. However, quantitative properties (e.g. continuous feature) are also important in biological processes, such as the concentration of proteins and the reaction rates. Thus, it is desirable to deal with both discrete and continuous features in the model (called *hybrid model*). From this fact, the next challenge is to apply the model checking approach to such hybrid models. Several hybrid models have been applied to biological pathway modeling, e.g. hybrid automata [6], *π*-calculus [14], ordinary differential equations (ODEs) [15], and hybrid functional Petri nets (HFPN) [16] and its extension (HFPNe) [17,18]. Among them, HFPNe and its related concepts have been accepted as a formal modeling method due to potential advantages of HFPNe possessing intuitive graphical representation and capabilities for mathematical analysis [19-23].

We use HFPNe to quantitatively model *C. elegans *vulval development mechanism, which best meets the features of biological processes. We have been developing an HFPNe based software "Cell Illustrator" [24,25] for modeling and simulating biological pathways [17,18,26]. It has been successfully employed to develop and analyze some pathway models for gene regulatory networks, metabolic pathways, and signaling pathways [16,27-31]. In this paper, Cell Illustrator is used as a software tool to model and simulate this complicated biological system in *C. elegans*.

The paper is organized as follows: In **Methods**, we first present an introduction of HFPNe and biological background of VPC fate determination mechanism. We demonstrate how to construct VPC fate model by using HFPNe afterwards. When determining cell fate from biological points of view, several biological fate determination rules can be considered. We thus employ two major biological fate determination rules – **Rule I **and **Rule II **– to the VPC fate model. Two rules are used to determine the cell fate from two viewpoints – temporal interval and temporal order – of time course expression of cell fate candidates. Next, we conduct 10,000 simulations for each of 48 sets of different genotypes which are the combination of four mutants and AC (anchor cell). The simulation procedures such as noise parameters, high-speed simulation engine, and the emulation of the temporal stimulations are also described. Finally, we examine the consistency and correctness of the VPC fate model, and evaluate proposed two rules by comparing with three simulation targets consisting of predicted fate patterns obtained from *in silico *and *in vivo *experiments. In **Results and Discussion**, our simulation results suggest that **Rule I **on the temporal interval is more reasonable than **Rule II **owing to the high coverage of predicted fate patterns (except for *lin-15ko; lin-12ko *double mutants), (ii) for the *lin-15ko; lin-12ko *double mutants, the coverage will be considerably augmented, if the number of animal population is increased in the *in vivo *experiments, and (iii) unmatched fate patterns of *lin-15ko *and *ac-; lin-15ko*, still have the possibility to be examined in *in vivo *experiments by enlarging animal numbers. More insights concerning the hybrid lineage are also suggested and discussed. The final section concludes the paper and addresses the contributions of the work.

## Methods

**Figure 1:**
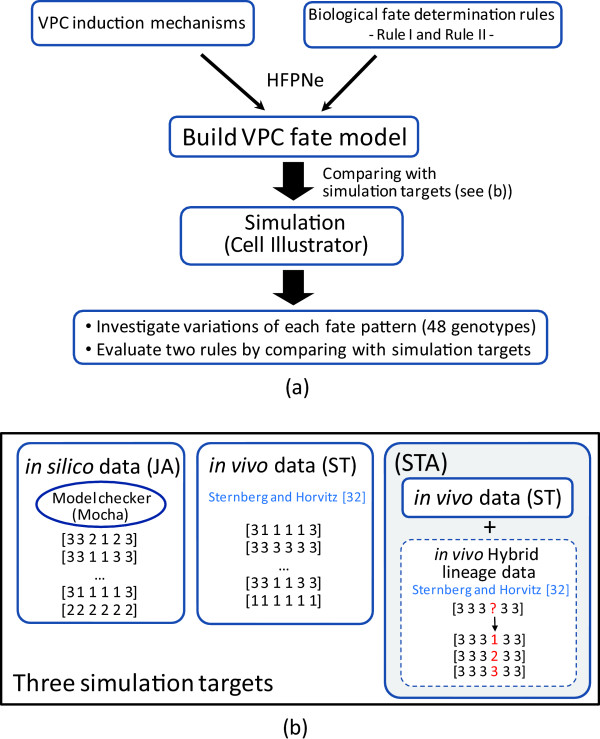
**(a) Procedure overview of the Methods section. (b) Schematic view of three simulation targets.**

Figure 1(a) illustrates the procedure overview of **Methods**. Briefly, starting from an introduction of HFPNe and VPC induction mechanisms, we first show the processes of constructing HFPNe-based VPC fate model (VPC fate model for short). We then employ two major biological fate determination rules to the VPC fate model from different viewpoints: temporal interval and temporal order. Finally, we execute 480,000 simulations in total for different genotypes by comparing with three simulation targets (i.e., **JA**, **ST**, and **STA**) (see Figure 1(b)). **JA **consists of fate patterns obtained by using model checking approach (with MOCHA); **ST **is the fate patterns summarized by Sternberg and Horvitz [32]; and **STA **is the fate patterns derived from [32] including hybrid lineage data. The aims of the simulation are: (i) to investigate predicted fate patterns variations of each genotype, and (ii) to evaluate two rules employed to VPC fate model.

### Modeling biological pathways with hybrid functional Petri net with extension (HFPNe)

#### Brief introduction of HFPNe: an enhanced Petri net for modeling biological interactions

Petri net is a network which consists of *place*, *transition*, *arc*, and *token*. A place can hold tokens as its content. A transition has arcs coming from places and arcs going out from the transition to some places. A transition with these arcs defines a firing rule in terms of the contents of the places where the arcs are attached [33].

Due to the limitation of conventional Petri net and more requirements in modeling, Matsuno *et al*. have defined *hybrid functional Petri net *(HFPN for short) in 2003 [16]. However, when modeling biological pathways, it has been noticed that several useful extensions should be applied for modeling and simulating more complicated biopathway processes (e.g., activities of enzymes for a multi-modification protein) and other biological processes that are not normally treated in biological pathways (e.g., alternative splicing and frameshifting) [18]. Therefore, Nagasaki *et al*. have proposed a new enhanced Petri net architecture *hybrid functional Petri net with extension *(HFPNe) in 2004. They have firstly used the new terminology in HFPNe to bridge the gap between the researchers of computer science and biology. In other words, the terms of place, transition, arc, and token are named as *entity*, *process*, *connector*, and *content *respectively.

**Figure 2:**
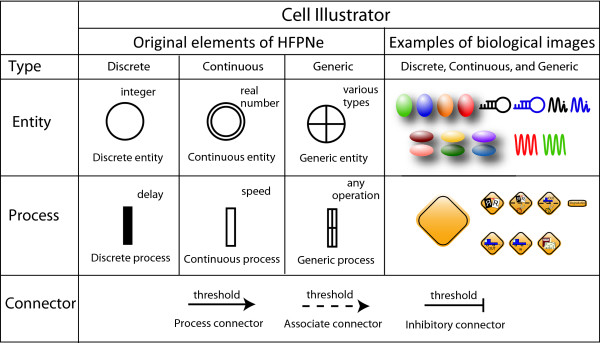
**Basic elements of HFPNe and examples of biological icons in Cell Illustrator**. In Cell Illustrator [24-26], HFPNe elements are replaced with the biological icons defined in the Cell System Ontology [47]. This replacement makes the HFPNe model of a biological pathway more comprehensible (see [17] for details).

HFPNe can deal with three types of data – discrete, continuous, and generic – and comprises three types of elements – entities, processes, and connectors – whose symbols are illustrated in Figure 2. A discrete entity holds a positive integer number of content. A discrete process is the same notion as used in the traditional discrete Petri net [33]. A continuous entity holds a nonnegative real number as concentration of a substance such as mRNA and protein. A continuous process is used to represent a biological reaction such as transcription and translation, at which the reaction speed is assigned as a parameter. A generic entity can hold various kinds of types including object, e.g., the string of nucleotide base sequence. A generic process can deal with any kind of operations (e.g., alternative splicing and frameshifting) to all types of entities. Connectors are classified into three types: process connector, associate connector, and inhibitory connector. Process connector connects an entity to a process or vice versa. Associate or inhibitory connector represents a condition and is only directed from an entity to a process. Each of process connector from an entity, associate connector, and inhibitory connector has a threshold by which the parameter assigned to the process at its head is controlled. A process connector from an entity or an associate connector (an inhibitory connector) can participate in activating (repressing) a process at its head, as far as the content of an entity at its tail is over the threshold. For either of associate or inhibitory connectors, no amount is consumed from an entity at its tail. The followed section only gives the mathematic definitions of HFPNe. For the detailed formal definition and properties of HFPNe, the readers are suggested to refer to [18].

#### Basic definitions of HFPNe

For modeling complex biological processes intuitively, we are required to deal with various kinds of biological information, e.g. the density of molecules, the number of molecules, sequences, molecular modifications, binding location, localization of molecules, etc. To cope with this feature in biological system modeling, we introduce *types *for biological entities and processes.

The set *T *of *types *is defined by the following abstract syntax:



Then, for *θ *∈ *T*, we define the *domain D*(*θ*) of *θ *as follows:

1. *D*(boolean) = {true, false}, *D*(integer) = **Z **(the set of integers), *D*(integer+) = **N **(the set of nonnegative integers), *D*(real) = **R **(the set of real numbers), *D*(real+) = **R**^≥**0 **^(the set of nonnegative real numbers), *D*(string) = **S **(the set of strings over some alphabet).

2. *D*(pair(*θ*_1_, *θ*_2_)) = *D*(*θ*_1_) × *D*(*θ*_2_).

3. *D*(list*θ*) = ∪_*k *≥ 0 _*D*(*θ*)^*k*^.

4. *D*(object(*θ*_1_, ⋯, *θ*_*n*_)) = *D*(*θ*_1_) × ⋯ × *D*(*θ*_*n*_).

For convenience, we denote D* = ∪_*θ *∈ *T*_*D *(*θ*).

Let *E *be a finite set. A *type function *for *E *is a mapping *τ*: *E *→ *T*. For *e *∈ *E*, *τ*(*e*) is called the *type *of *e*. A *marking *of *E *is a mapping *M *: *E *→ D* satisfying *M*(*e*) ∈ *D*(*τ*(*e*)) for *e *∈ *E*. For *e *∈ *E*, *M*(*e*) called the *mark *of *e*. We denote by ℳ the set of all markings of *E*. We can regard ℳ as the set ∏_*e*∈*E *_*D*(*τ*(*e*)).

Consider a function *f *: ℳ → **R**. For a subset *F *⊆ *E *and an element *v *∈ ∏_*e *∈ *F *_*D*(*τ*(*e*)), let *f *[*F *= *v*]: ∏_*e *∈ *E*-*F *_*D*(*τ*(*e*)) → **R **be the function obtained from *f *by restricting the value for *F *to *v*, i.e. *f *[*F *= *v*](*z*) = *f*(*z*, *v*) for *z *∈ ∏_*e *∈ *E*-*F *_*D*(*τ*(*e*)). Let *F *be a subset of *E *such that *e *∈ *F *satisfies *D*(*τ*(*e*)) = **R **or **R**^≥ **0**^. We say that the function *f *is *continuous for F *if *f *[*E *- *F *= *v*]: ∏_*e *∈ *F *_*D*(*τ*(*e*)) → **R **is continuous on ∏_*e *∈ *F *_*D*(*τ*(*e*)) for any *v *∈ ∏_*e *∈ *E*-*F *_*D*(*τ*(*e*)).

Based on the above terminology, we define the notion of hybrid functional Petri net with extension (HFPNe). The basic idea of HFPNe is two-fold. The first is to introduce types with which we can deal with various data types. The second is to employ functions of marking *f*(*M*) to determine the weight, delay, and speed, etc. which control the system behavior. In the following definition, we use different names instead of place, transition, arc, etc. which are conventionally used in Petri net theory since biological system modeling requires more intuitive names for representing biological entities and processes.

**[Definition 1] **We define a *hybrid functional Petri net with extension *(HFPNe) *H *= (*E*, *P*, *h*, *τ*, *C*, *d*, *α*) as follows:

1. *E *= {*e*_1_, ⋯, *e*_*n*_} is a non-empty finite set of *entities *and *P *= {*p*_1_, ⋯, *p*_*m*_} is a non-empty finite set of *processes*, where we assume *E *∩ *P *= ∅.

2. *h *: *E *∪ *P *→ {discrete, continuous, generic} is a mapping called the *hybrid function*. Terms "discrete" and "continuous" correspond to those in hybrid Petri net [16] and "generic" is a newly introduced name which can be of any type in *T*. A process *p *∈ *P *with *h*(*p*) = discrete (resp., continuous, generic) is called a *discrete process *(resp., *continuous process*, *generic process*). An entity *e *∈ *E *with *h*(*e*) = discrete (resp., continuous, generic) is called a *discrete entity *(resp., *continuous entity*, *generic entity*).

3. *τ*: *E *→ *T *is a type function for *E *such that *τ*(*e*) = integer+ if *e *is a discrete entity, and *τ*(*e*) = real+ if *e *is a continuous entity.

4. *C *= (*EP*, *PE*, *a*, *w*, *u*) consists of subsets *EP *⊆ *E *× *P *and *PE *⊆ *P *× *E*. An element in *EP *∪ *PE *is called a *connector*. Each connector has a *connector type *which is given by a mapping *a *: *EP *∪ *PE *→ {process, associate, inhibitor} called the *connector type function *which satisfies the conditions: (i) *a*(*c*) = process for *c *∈ *PE*. (ii) All connectors *c *= (*e*, *p*) ∈ *EP *satisfy the conditions in Table 1(a) and all connectors *c *= (*p*, *e*) ∈ *PE *satisfy the conditions in Table 1(b). A connector *c *= (*e*, *p*) ∈ *EP *is called a *process connector *(resp., an *associate connector*, an *inhibitory connector*) if *a*(*c*) = process (resp., associate, inhibitor). "Process connector", "associate connector" and "inhibitory connector" correspond to *normal arc*, *test arc *and *inhibitory arc*, respectively. For a connector *c *= (*p*, *e*) ∈ *PE*, *a*(*c*) = process by definition and we also call it a *process connector*. We say that a connector *c *= (*e*, *p*) ∈ *EP *is *discrete *(resp., *continuous*, *generic*) if *p *is a discrete process (resp., continuous process, generic process). In the same way, we also say that *c *= (*p*, *e*) ∈ *PE *is *discrete *(resp., *continuous*, *generic*) if *p *is a discrete process (resp., continuous process, generic process). Let ℳ be the set of all markings of *E *and let *F *be the set of continuous entities in *E*. Then we denote  = {*f *|*f *: ℳ → **N**},  = {*f *|*f *: ℳ → **R**^≥ **0 **^is continuous for *F*},  = {*f *|*f *: ℳ → D*}, and  = {*f *|*f *: ℳ → {true, false}}.

**Table 1:** The conditions of the connector type

	connector type		process connector			associate or inhibitory connector		
	
		process type	discrete	continuous	generic	discrete	continuous	generic
	
(a)	entity	discrete	X	-	X	X	X	X
	
	type	continuous	X	X	X	X	X	X
	
		generic	-	-	X	X	X	X
	connector type		process connector			associate or inhibitory connector		
	
		process type	discrete	continuous	generic	discrete	continuous	generic
	
(b)	entity	discrete	X	-	X	-	-	-
	
	type	continuous	X	X	X	-	-	-
	
		generic	X	X	X	-	-	-

Table 1: (a) For a connector *c *= (*e*, *p*) ∈ *EP*, the entity type *h*(*e*), the process type *h*(*p*) and the connector type *a*(*c*) must satisfy the following conditions, where X means that the connection is allowed and – means that the connection is not allowed. (b) For a connector *c *= (*p*, *e*) ∈ *PE*, the connector type *a*(*c*) is process by definition. The entity type *h*(*e*) and the process type *h*(*p*) must satisfy the following conditions, where X means that the connection is allowed and – means that the connection is not allowed.

Then *w *and *u *are given as follows:

(a) *w *:  is a function called the *activity function *such that for a connector *c *∈ *EP *(i) *w*(*c*) ∈  if *c *is discrete, (ii) *w*(*c*) ∈  if *c *is continuous, (iii) *w*(*c*) ∈  if *c *is generic. For a connector (*e*, *p*), *w*(*e*, *p*) be used as a function giving the threshold in discrete and continuous cases and the condition in generic case which is required for enabling the process *p*.

(b) *u *:  is a function called the *update function *which satisfies the following conditions: For a connector *c *∈ *EP *∪ *PE*, let *c *= (*e*, *p*) ∈ *EP *or *c *= (*p*, *e*) ∈ *PE*. (i) *u*(*c*) ∈  if *c *is discrete. (ii) *u*(*c*) ∈  if *c *is continuous. (iii) If *c *is generic, then *u*(*c*) is a function in  such that *u*(*c*)(*M*) is in *D*(*τ*(*e*)) for any marking *M *∈ ℳ. For a connector *c *= (*e*, *p*) or *c *= (*p*, *e*), *u*(*c*) is used as a function which will update the mark of *e*.

5. *d *: *P*_discrete _→  is a mapping called the *delay*, where *P*_discrete _is the set of discrete processes in *P*. For a discrete process *p*, *d*(*p*): ℳ → **R**^≥**0 **^is called the *delay function *of *p*.

6. *α *> 0 is a real number called the *generic time*. The generic time is used as the clock for generic processes.

We introduce a parameter *t *∈ **R**^≥ **0 **^called the *time *to a hybrid functional Petri net with extension *H *= (*E*, *P*, *h*, *τ*, *C*, *d*, *α*). Given a marking *I *called the *initial marking*, we define a marking *M*(*t*) called the *marking at time t *and a marking *M*_*r*_(*t*) called the *reserved marking at time t *for *t *≥ 0 in the following way.

By convention, we denote *M*(*e*, *t*) = *M*(*t*)(*e*) and *M*_*r*_(*e*, *t*) = *M*_*r*_(*t*)(*e*) for *e *∈ *E*. We define (*t*) by (*e*, *t*) = *M*(*e*, *t*) - *M*_*r*_(*e*, *t*) for discrete and continuous entities and (*e*, *t*) = *M*(*e*, *t*) for generic entities *e*.

First, we define *M*(0) = *I*, *M*_*r*_(*e*, 0) = 0 for all discrete and continuous entities *e*. For all generic entities *e*, *M*_*r*_(*e*, *t*) = null (the empty list) for any *t *≥ 0. For *t *> 0, we define *M *(*t*) and *M*_*r*_(*t*) in the following way.

For a process *p *∈ *P *at time *t*, if the following conditions are satisfied, then the process *p *is said to be *enabled *at time *t*. Otherwise the process is said to be *disabled *at time *t*.

1. If *p *is a discrete process, then for all connectors *c *= (*e*, *p*) ∈ *EP *the following conditions hold:

(a) (*e*, *t*) ≥ *w*(*e*, *p*)(*M*(*t*)) if *a*(*c*) ≠ inhibitor.

(b) (*e*, *t*) <*w*(*e*, *p*)(*M*(*t*)) if *a*(*c*) = inhibitor.

2. If *p *is a continuous process, then for all connectors *c *= (*e*, *p*) ∈ *EP *the following conditions hold:

(a) (*e*, *t*) ≥ *w*(*e*, *p*)(*M*(*t*)) if *a*(*c*) ≠ inhibitor.

(b) (*e*, *t*) ≤ *w*(*e*, *p*)(*M*(*t*)) if *a*(*c*) = inhibitor.

3. If *p *is a generic process, then for all connectors *c *= (*e*, *p*) ∈ *EP *the following conditions hold:

(a) *w*(*e*, *p*)((*t*)) = true if *a*(*c*) ≠ inhibitor.

(b) *w*(*e*, *p*)(*t*)) = false if *a*(*c*) = inhibitor.

If a disabled process turns to be enabled at time *t*, the process is said to be *triggered *at time *t*. If an enabled process turns to be disabled or a disabled process turns to be enabled at time *t*, the process is said to be *switched *at time *t*. If a discrete process *p *is triggered at time *t*, we say that the discrete process can be *fired *at time *t *+ *d*(*p*)(*M*(*t*)). If a generic process *p *is triggered at time *t*, we say that the generic process can be *fired *at time *t *+ *α*.

For an entity *e *∈ *E *and time *t*, let *S*_*d*_(*t*) be the set of discrete processes which can be fired at time *t*, and let *U*_*d*_(*t*) be the set of discrete processes which are triggered at time *t*. For a discrete process *p *that can be fired at time *t*, we denote by *q*(*p*, *t*) the time when *p *is triggered. Let *S*_*c*_(*t*) be the set of continuous processes which are enabled at time *t*. Let *S*_*g*_(*t*) be the set of generic processes which can be fired at time *t*.

Note that we can choose a sufficiently small *ε*_*t *_> 0 such that in the interval [*t *- *ε*_*t*_, *t*), neither discrete nor generic process is triggered or can be fired and no continuous process is switched.

Also note the following facts:

1. *S*_*c*_(*t *- *ε*_*t*_) = *S*_*c*_(*t'*) for any *t' *∈ [*t *- *ε*_*t*_, *t*) since no continuous process is switched in the interval [*t *- *ε*_*t*_, *t*).

2. (*t'*) is constant on *E *- *E*_continuous _in the interval [*t *- *ε*_*t*_, *t*) since neither discrete nor generic process is triggered or can be fired in the interval [*t *- *ε*_*t*_, *t*), where *E*_continuous _= {*e *∈ *E *|*e *is continuous}.

3. For any continuous connector *c*, *u*(*c*)((*t'*)) is continuous on [*t *- *ε*_*t*_, *t*) since by definition *u*(*c*) is continuous for *E*_continuous _and (*t'*) is constant on *E *- *E*_continuous _in the interval [*t *- *ε*_*t*_, *t*).

Then *M*(*t*) is defined by the following procedure:

1. *Tmp *← *M*(*t *- *ε*_*t*_), *Tmp*_*r *_← *M*_*r*_(*t *- *ε*_*t*_)

2. **if ***t *= *αk *for some integer *k *≥ 1 **then**

   **for each **generic process *p *∈ *S*_*g*_(*t*)

      *Tmp' *← *Tmp*

      **for each **(*e*, *p*) ∈ *EP *with *a*(*e*, *p*) = process

         *Tmp'*(*e*) ← *u*(*e*, *p*)(*Tmp*)

      **for each **(*p*, *e*) ∈ *PE*

         *Tmp'*(*e*) ← *u*(*p*, *e*)(*Tmp*)

      *Tmp *← *Tmp'*

3. **for each **continuous process *p *∈ *S*_*c*_(*t *- *ε*_*t*_)

      *Tmp' *← *Tmp*

      **for each **(*e*, *p*) ∈ *EP *with *a*(*e*, *p*) = process

         

   **for each **(*p*, *e*) ∈ *PE*

         

      *Tmp *← *Tmp'*

4. **for each **discrete process *p *∈ *S*_*d*_(*t*)

      *Tmp' *← *Tmp*

      **for each **(*e*, *p*) ∈ *EP *with *a*(*e*, *p*) = process

         *Tmp'*(*e*) ← *Tmp'*(*e*) - *u*(*e*, *p*)((*q*(*p*, *t*)))

      **for each **(*p*, *e*) ∈ *PE*

         *Tmp'*(*e*) ← *Tmp'*(*e*) + *u*(*p*, *e*)((*q*(*p*, *t*)))

      *Tmp *← *Tmp'*

5. *M*(*t*) ← *Tmp*

Then *M*_*r*_(*t*) is defined as follows:

6. **for each **entity *e *with *h*(*e*) = discrete or continuous

   

7. *M*_*r*_(*t*) ← *Tmp*_*r*_.

We call *M*(*t*) (*t *≥ 0) the *behavior *of *H *starting at the initial marking *M*(0) = *I*.           □

In the next section, we will introduce the biological background and explain the modeling method of *C. elegans *vulval development mechanisms.

### Biological background and modeling of *C. elegans *vulval development

#### Biological background of *C. elegans *vulval development

**Figure 3:**
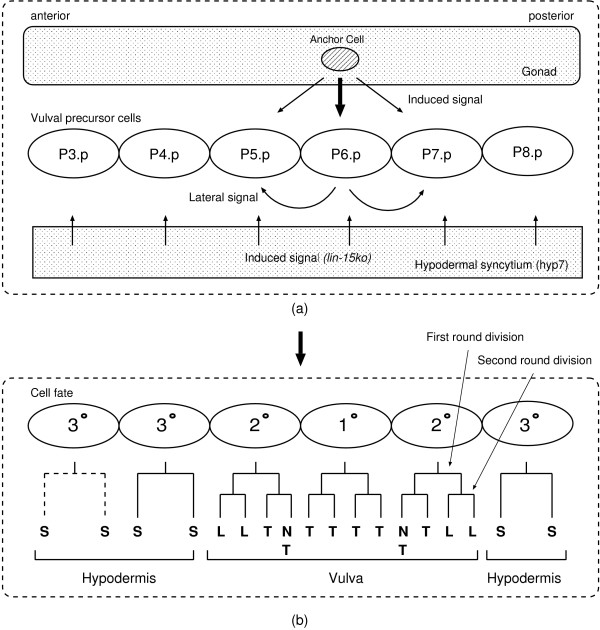
**Schematic representations of VPC fate specification in wild-type hermaphrodites**. (a) The anchor cell produces a graded inductive signal and causes six equivalent cells to adopt fates in a precise pattern. (b) The sublineage is generated according to each specified cell fate. The sublineage is a determined pattern of cell divisions which produces a characteristic set of progeny cell types. Sternberg and Horvitz have defined vulval cell types (after two rounds of VPC divisions) by two criteria as follows: the axis of the third round nuclear divisions (L, longitudinal axis; T, transverse axis; N, no division; S is to join the large hypodermal syncytium (hyp7)), and adherence to the ventral cuticle [32].

The *C. elegans *vulva is an egg-laying organ which is constituted by the descendants of three VPCs. The three VPCs are the members of six initially equivalent VPCs that are consecutively numbered P3.p – P8.p (termed Pn.p cells). In response to extracellular signaling pathways, each VPC has a potential to adopt one of three alternative cell fates (1°, 2°, 3°) (see Figure 3(a)). Six cell fates of Pn.p cells comprise a cell fate pattern in the form of [P3.p P4.p P5.p P6.p P7.p P8.p]. In wild-type worms, P3.p – P8.p always adopt a same pattern of fates (i.e., [332123]). The sublineage is then generated according to each specified VPC fate. The sublineage is a determined pattern of cell divisions which produces a characteristic set of progeny cell types. Figure 3(b) shows the sublineage of respective VPC fate according to the criteria defined by Sternberg and Horvitz [32].

**Figure 4:**
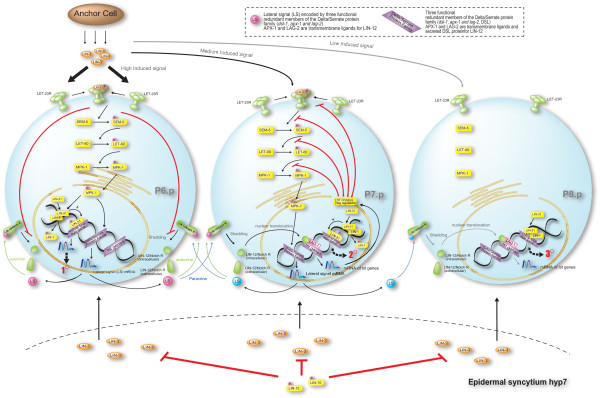
**Biological diagram for the multiple signaling events underlying VPC specification**. Biological diagram for the multiple regulatory signaling pathways underlying VPC fate specification. Three VPCs (they can be P6.p, P7.p and P8.p, or P6.p, P5.p and P4.p) according to the relative distance to the AC are selected to depict the signaling crosstalk. In the rightmost cell, the EGF/MAPK pathway cannot be activated because the induced signal (indicated by a grey line) received by LET-23, is lower than the threshold for induction, and the VPC hence adopts the 3° fate. The induction signal with high concentration (indicated by a heavy black line) activates the EGF/MAPK pathway and causes the 1° fate. It also has been known that two transcription factors, LIN-31 and LIN-1 are likely to be the downregulation targets of the MAPK pathway [42]. Both LIN-31 and LIN-1 can be phosphorylated by MAPK kinase MPK-1. LIN-31 and LIN-1 usually form a complex that is disrupted by the phosphorylation of LIN-31, and LIN-1 dissociates from LIN-31 to let LIN-31 play a role in the proper specification of VPCs. On the other hands, ligands for LIN-12 are members of the "DSL" family, an acronym derived from canonical ligands from *Drosophila *(*D*elta, *S*errate) and *C. elegans *(*L*AG-2). Binding of DSL ligands to LIN-12/Notch leads to the shedding of the LIN-12/Notch ectodomain (extracellular domain) via cleavage. The remaining transmembrane protein is cleaved constitutively, and the intracellular domain translocates to the nucleus, binding to the LAG-1 that usually exists as a transcription factor to repress *lst *genes. With the binding to LAG-1, *lst *genes expresses LST proteins to counteract the operations of EGF/MAPK pathways by inhibiting VPCs from becoming the 1° fate. For the details of LIN-12/Notch signaling in *C. elegans*, the readers are suggested to refer to [34].

In wild-type *C. elegans*, an inductive signal LIN-3, an EGF-like signal produced by gonad, activates the EGFR homolog LET-23 and a canonical Ras/MAPK cascade in P6.p adopting the 1° fate. In response to the inductive signal, P6.p produces LIN-12-mediated lateral signals (LS for short) that counteract the inductive signal from AC in two neighboring VPCs, which causes two VPCs to adopt the 2° fate. In other words, LS induces the expression of negative regulators (collectively termed *lateral signal target *(*lst*) genes [34]) against the EGF/Ras/MAPK pathway. LS is encoded by three functionally redundant members of the Delta/Serrate protein family (*dsl-1*, *apx-1*, and *lag-2*) and transduced by the LIN-12/Notch receptor. Each *lst *gene contains a cluster of binding site LBSs for LAG-1 that is a DNA binding protein forming a complex with the LIN-12 intracellular domain to activate the transcription of the target genes [34-36]. Furthermore, two functionally redundant synthetic Multivulva (*synMuv*) genes transcriptionally repress the target gene of *lin-3 *in the hypodermis, i.e., *synMuv *genes prevent the surrounding hypodermal syncytium hyp7 from generating inductive LIN-3 signals [37]. Without the activation of either inductive or lateral signaling pathways, P3.p, P4.p, and P8.p adopt the 3° fate [35]. Thus, the fates of 1°, 2°, and 3° are the productions of the coordination regulated by three signaling pathways, i.e., AC induced signaling pathways, the lateral signaling pathways, and the signaling pathways induced from hyp7 [32,35,38-41]. Figure 4 illustrates the biological diagram for the multiple signaling events underlying the VPC fate specification.

#### Modeling VPC fate specification mechanisms with HFPNe based on literature

**Figure 5:**
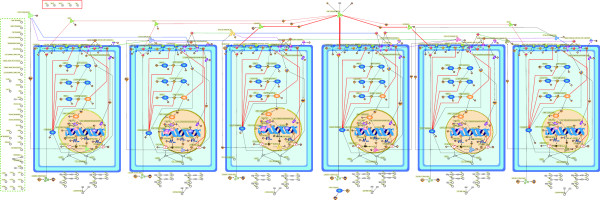
**The whole HFPNe based VPC fate model underlying the fate specification mechanisms involving six equivalent VPCs**.

Figure 5 exhibits a whole HFPNe based VPC fate model that is constructed by compiling and interpreting the information appeared in the literature concerning the VPC fate specification mechanisms [9,32,34-38,42-46]. The whole VPC fate model totally includes 427 entities, 554 processes and 780 connectors. The elements of HFPNe are changed to biological icons in Cell Illustrator (on the right side of Figure 2). The icons have been defined with one of the biological ontology information, called Cell System Ontology [47]. Although these changes have no effect on mathematical meaning, it is helpful for biologists to understand the pathways.

**Figure 6:**
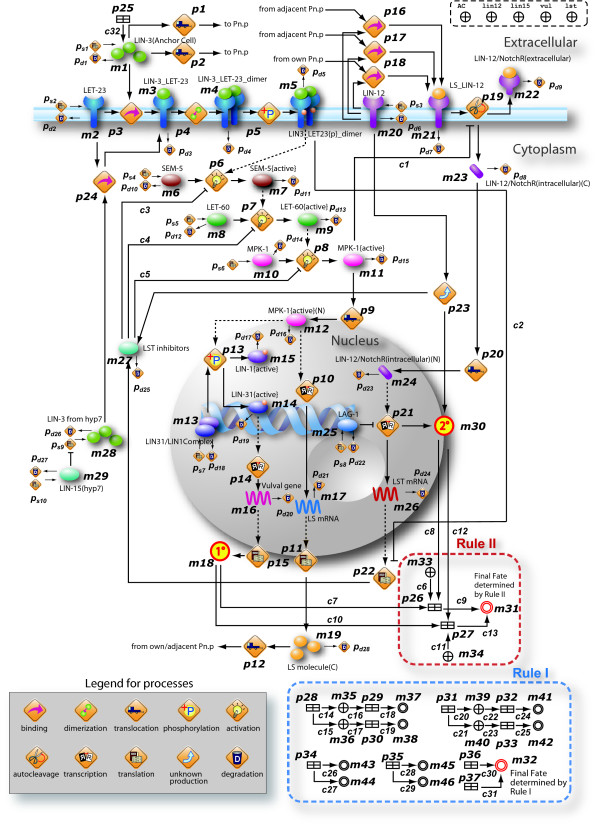
**HFPNe model of a single VPC in Figure 5**. The naming rules of the VPC fate model are defined as follows: (i) each "entity" is labeled with the name of a substance (e.g. LIN-3, LET-60, and *lst *gene), (ii) the name of a complex consisting of two or more protein components *A*_1_, *A*_2_, ⋯, *A*_*M *_(*M *∈ *Z*^+^) is represented as *A*_1 __*A*_2_ _⋯ *_A*_*M*_, and (iii) an additional label (C) or (N) is attached at the end of a substance name, when it happens to distinguish the location of the substance in the cytoplasm or the nucleus. The label of {active} denotes the active state of the enzyme, and {p} denotes that the substance is phosphorylated.

Since six VPCs are initially equivalent, we hereafter explain modeling operations by using a single VPC fate model as shown in Figure 6 whose six copies are embedded in the whole VPC fate model in Figure 5. In Figure 6, 24 events directly attending VPC fate specification are assigned to the processes *p*_*i *_∈ {*p*1, ⋯, *p*_24_} (see Table 2). A set of sink processes  denotes the natural degradation of the substances, whereas a set of source processes  denotes the translation reactions of initial entities. The left 13 processes {*p*_25_, *p*_26_, ⋯, *p*_37_} are generic processes, which contribute to play the roles of extracellular stimuli ({*p*25}) and the cell fate specification ({*p*_26_, *p*_27_, ⋯, *p*_37_}). Variable *m*_*x *_∈ {*m*_1_, ⋯, *m*_32_} denotes the concentration of corresponding substance (see Table 3).

**Table 2:** Biological interpretation based on literature and assignment of each process in Figure 6

Wet experiments results published in literature	#1	#2	Reaction type	Refs
Translocation of LIN-3 emanated from AC to P5.p and P7.p	***p*_1_**	*LSMass*(*m*_1_*0.1*low, 0.1)	Translocation	[9,35]
Translocation of LIN-3 emanated from AC to P3.p, P4.p and P8.p	***p*_2_**	*LSMass*(*m*_1_*0.1*mid, 0.1)	Translocation	[9,35]
ligands LIN-3 binding to LET-23 to form a ligand-receptor complex	***p*_3_**	*LSMass*(*m*_1_*0.1*high, 0.1)	Binding	[43,44]
Two identical LIN-3 LET-23 complex combining to form a dimer	***p*_4_**	*LSMass*(*m*_3_*0.1, 0.5)	Dimerization	[43,44]
Autophosphorylation following the dimerization of ligand-receptor complex	***p*_5_**	*LSMass*(*m*_4_*0.1, 0.5)	Autophosphorylation	[43,44]
SEM-5 is activated by LIN-3 LET-23 dimer	***p*_6_**	*LSMass*(*m*_5_*m_6_*0.1, 0.5)	Enzymic reaction	[9,35]
LET-60 is activated by upstream SEM-5	***p*_7_**	*LSMass*(*m*_7_*m_8_*0.1, 0.5)	Enzymic reaction	[9,35]
Inactive MPK-1 is activated by upstream LET-60	***p*_8_**	*LSMass*(*m*_9_*m_10_*0.1, 0.5)	Enzymic reaction	[9,35]
The movement of MPK-1 from cytoplasm to nucleus	***p*_9_**	*LSMass*(*m*_11_*0.1, 0.5)	Translocation	-
Active MPK-1(N) downregulates the target genes of *lst *and transcribes the mRNA of lateral signal (LS)	***p*_10_**	*LSMass*(*m*_12_*0.1, 0.5)	Transcription	[9]
LS mRNA is translated to LS molecules	***p*_11_**	*LSMass*(*m*_17_*0.1, 0.5)	Translation	-
Translated LS molecules are released to combine with	***p*_12_**	*LSMass*(*m*_19_*0.1, 0.5)	Translocation	[34]
LIN-12 receptors				
LIN-31/LIN-1 complex is dissociated to individual active	***p*_13_**	*LSMass*(*m*_12_*m_13_*0.1, 0.5)	Phosphorylation	[38,45]
LIN-31 and LIN-1 by the phosphorylation of active MPK-1				
Active a MPK-1(N) acts as transcription factor to tran-	***p*_14_**	*LSMass*(*m*_14_*0.1, 0.5)	Transcription	[42]
scribe vulval genes to mRNA				
mRNA of vulval genes is translated and cause the 1	***p*_15_**	*LSMass*(*m*_16_*0.1, 0.5)	Translation	[42]
cell fate				
LIN-12 receptor received the LS molecules from the ad-	***p*_16_**	*LSMass*(*m*_20_*m_κ_*1.0, 0.1)	Binding	[32,34]
jacent Pn.p and shape a ligand-receptor complex				
LIN-12 receptor received the LS molecules from its own	***p*_17_**	*LSMass*(*m*_20_*m_κ_*1.0, 0.1)	Binding	[32,34]
Pn.p and shape a ligand-receptor complex				
LIN-12 receptor received the LS molecules from the ad-	***p*_18_**	*LSMass*(*m*_20_*m_κ_*0.1, 0.1)	Binding	[32,34]
jacent Pn.p and shape a ligand-receptor complex				
Binding of LS ligands to LIN-12/Notch receptor leads to	***p*_19_**	*LSMass*(*m*_21_*0.1, 0.5)	Shedding/Cleavage	[34]
shedding of the LIN-12/Notch extracellular domain via cleavage				
Cleaved intracellular domain of LIN-12/Notch receptor move from cytoplasm to nucleus	***p*_20_**	*LSMass*(*m*_23_*0.1, 0.5)	Translocation	[34,46]
Cleaved LIN-12/Notch receptor promote the target *lst *genes transcribed into mRNA of *lst *genes	***p*_21_**	*LSMass*(*m*_24_*0.1, 0.5)	Transcription	[34,36]
LST mRNA is translated to LST in cytoplasm	***p*_22_**	*LSMass*(*m*_26_*0.1, 0.5)	Translation	-
LIN-12 immediately induces *lst *expression thus prevents cells from engaging the mechanisms reducing LIN-12 activity	***p*_23_**	*LSMass*(*m*_20_*lin12_*init*, 0.1)	Production	[9]
LIN-3 emanating from hyp7 binds to LET-23 to form a complex	***p*_24_**	*LSMass*(*m*_2_*m_28_*0.1, 0.5)	Expression	[9,37]

Table 2: The column of #1 represents corresponding processes in the HFPNe model. Twenty-four events are assigned to the processes *p*_*i *_∈ {*p*_1_, ⋯, *p*_24_}. Each reaction speed of the processes is assigned as shown in the column of #2, in which several reaction speeds have been tuned manually. Reaction types of the processes are described in the fourth column with the literature facts given in the fifth column. Variable *m*_*x *_∈ {*m*_1_, ⋯, *m*_32_} denotes the concentration of corresponding substance (see Table 3). The variable *mk *is used to collectively denote the concentration of the LS molecules generated from the adjacent Pn.p. The values of *high, mid*, and *low *are assigned to 100, 1, and 0.01. For example, the process ***p***_9 _has a reaction speed with a noise denoted by *LSMass*(*m*_11 _*0.1, 0.5), i.e., the reaction speed depends on the concentration of MPK-1{active} (C) in the cytoplasm (*m*_11_). *LSMass*() is a function of log-normal distribution (see text).

**Table 3:** Entities in the HFPNe model of Figure 6

Entity Name	Variable (*m*_*x*_)
LIN-3(AC)	*m*_1_
LET-23	*m*_2_
LIN-3/LET-23 complex	*m*_3_
LIN-3/LET-23 dimer	*m*_4_
LIN-3/LET-23 dimer{p}	*m*_5_
SEM-5	*m*_6_
SEM-5{active}	*m*_7_
LET-60	*m*_8_
LET-60{active}	*m*_9_
MPK-1	*m*_10_
MPK-1{active}(C)	*m*_11_
MPK-1{active}(N)	*m*_12_
LIN-1/LIN-31 complex	*m*_13_
LIN-31{active}	*m*_14_
LIN-1{active}	*m*_15_
Vulval gene mRNA	*m*_16_
LS mRNA	*m*_17_
1° cell fate	*m*_18_
LS molecules(C)	*m*_19_
LIN-12/Notch receptor	*m*_20_
LIN-12R/LS complex	*m*_21_
Cleaved fraction of LIN-12R/LS (extracellular domain)	*m*_22_
Intracellular domain of LIN-12/LS complex (C)	*m*_23_
Intracellular domain of LIN-12/LS complex (N)	*m*_24_
LAG-1	*m*_25_
LST mRNA	*m*_26_
LST inhibitors	*m*_27_
LIN-3 emanating from hyp7	*m*_28_
LIN-15 in hyp7	*m*_29_
2° cell fate	*m*_30_
Final fate determined by Rule II	*m*_31_
Final fate determined by Rule I	*m*_32_

Table 3: Variable *m*_*x *_∈ {*m*_1_, ⋯, *m*_32_} indicates the concentration of each substance that actually takes part in the biological reactions. Initial value represents the initial content of an entity.

The modeling operation starts from the source process  denoting an activity that the substance takes part in the reaction. Here we only explain the case of Ras/MAPK inductive signaling pathway: LIN-3 ligands and corresponding receptors LET-23 are assigned to the entities (*m*_1 _and *m*_2_) connected from the source processes ( and ), respectively. Next, the ligand-receptor binding reaction where two entities (*m*_1 _and *m*_2_) merge into an entity *m*_3 _denoting ligand-receptor complex is represented via the process *p*_3_. Since two ligand-receptor complexes shape a dimer, the process *p*_4 _is used to represent the homodimerization connecting from the input entity *m*_3 _as well as connecting to the output entity *m*_4 _of LIN-3/LET-23 dimer. Note that the stoichiometry of the input connector of *p*_4 _is set to 2 due to the dimerization. The dimer is autophosphorylated subsequently which is modeled by using a phosphorylation process *p*_5 _connecting from and to the entities of *m*_4 _and *m*_5_, respectively. Succedent reactions for the products of the canonical cascades: active SEM-5 (*m*_7_), LET-60 (*m*_9_), and MPK-1 (*m*_11_) are modeled in the same way using the processes of activation (*p*_6_, *p*_7 _and *p*_8_). Activated MPK-1 then moves from the cytoplasm to the nucleus. This movement is modeled as a translocation reaction by *p*_9_.

Based on the understanding of the literature and the hypothesis reported in [9], we first model downstream regulation of Ras/MAPK cascades and succedent intracellular reactions induced by LIN-12/Notch signaling according to newly published literature. The details of the new biological facts are described in the caption of Figure 4. The new facts concerning the mechanisms of the downstream regulation of Ras/MAPK cascades are modeled as follows: The phosphorylation by *m*_12 _further disrupts the formation of LIN-31/LIN-1 complex (*m*_13_) which applies the process of phosphorylation (*p*_13_). The processes *p*_14 _and *p*_15 _are used to model the transcription of target genes and the translation of mRNA (*m*_16_) regulated by LIN-31 (*m*_31_) that acts as a transcriptional activator promoting the 1° fate as a fate candidate (*m*_18_).

### Two biological fate determination rules applied to VPC fate model

When determining cell fates from biological points of view, several biological fate determination rules can be taken into account. One major biological fate determination rule (**Rule II**) based on temporal order has been applied to a qualitative model by Fisher *et al*. [9]. The approach is based on a discrete model. However, the other rules such as temporal interval based one (**Rule I**) cannot be handled in the same discrete model. This is because the model cannot deal with the quantitative properties (e.g., continuous feature) that are also important to biological processes, such as the concentration of proteins and reaction rates. With inspiring by this limitation of qualitative models, we employ these two major biological fate determination rules – **Rule I **and **Rule II **– to VPC fate model. The model has both discrete and continuous features.

**Figure 7:**
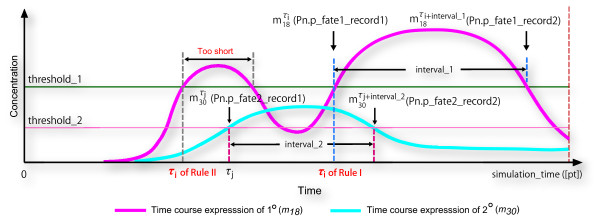
**A schematized diagram depicting the fate specification methods by using Rule I and Rule II**. "*τ*_*i *_of Rule I" represents the first time epoch that the concentration of 1° (*m*_18_) exceeds *threshold_*1, and the time length *interval_*starting from "*τ*_*i *_of Rule I" is longer than or equal to the given *interval *when using **Rule I**. "*τ*_*j *_of Rule II" represents the first time epoch that the concentration of 1° exceeds *threshold_*1, no matter how long this interval will continue when using **Rule II**. *τ*_*i *_represents the first time epoch that the concentration of 2° (*m*_30_) exceeds *threshold_*2. Variables *m*_18 _(denoting the concentration of 1°) and *m*_30 _(denoting the concentration of 1°) are regarded as two cell fate candidates for determining final VPC fate. *Pn.p_fate*1/2_*record*1/2 indicated in the brackets are the entity names used in Cell Illustrator.

Two fate determination rules are as follows: (i) For **Rule I**, the fate will be determined if it satisfies the conditions that (1) the fate can sustain the behaviors at a certain over-threshold state within a given length of time, and (2) the time epoch of the certain over-threshold in (1) is earlier than the other fate candidate. Figure 7 shows a schematized diagram of a simulation result. In Figure 7, the cell fate is determined to the 2° fate by using **Rule I**. The time course expression of 1° is maintained at the over-threshold state during *interval_*1, and that of 2° is maintained at the over-threshold state during *interval_*2. Although the time course expression of 1° has two over-threshold states, only the second interval *interval*_1 is longer than the given *interval*. Thus, the time epoch labeled with "*τ*_*i *_of Rule I" is regarded as the initial time epoch inducing the second over-threshold state. The VPC adopts the 2° fate due to *τ*_*j *_<"*τ*_*i *_of Rule I".

(ii) For **Rule II**, the cell fate will be priorly adopted according to the temporal sequence of the first time epoch inducing over-threshold state. In Figure 7, we can observe that the first time epoch (i.e., "*τ*_*i *_of Rule II") inducing over-threshold state of 1° is earlier than 2°, and therefore the 1° fate will be adopted by **Rule II **no matter how long the over-threshold state of 1° will continue. Preliminary notations and mathematical definitions of two rules are given in the Additional file 1.

In Figure 6, two dashed-line ellipses illustrate the topological connections representing **Rule I **(blue ellipse) and **Rule II **(red ellipse) which jointly use the values of *m*_18 _and *m*_30 _as the inputs for the fate specification. Variables *m*_18 _(denoting the concentration of 1°) and *m*_30 _(denoting the concentration of 2°) are regarded as two cell fate candidates for determining final VPC fate. Two rules are implemented with a script-based language Pnuts in Cell Illustrator [48]. The detailed properties and descriptions of the entities concerning the fate specification are summarized in Table 4. The activity functions of related processes and update functions of the connectors are summarized in Tables 5 and 6.

**Table 4:** Properties of entities for the HFPNe model of Figure 6

Variable	Entity name	Entity type	Value type	Initial value	Variable description
AC	AC	Generic	Boolean	true/false	Entity can be switched according to the genotype
lin12	lin-12	Generic	String	"wt"/"ko"/"gf"	Entity can be switched according to the genotype
lin15	lin-15	Generic	Boolean	true/false	Entity can be switched according to the genotype
vul	vul	Generic	Boolean	true/false	Entity can be switched according to the genotype
lst	lst	Generic	Boolean	true/false	Entity can be switched according to the genotype
*m*_31_	Pn.p_FinalFateII	Continuous	Double	0	Final Fate determined by Rule II
*m*_32_	Pn.p_FinalFateI	Continuous	Double	0	Final Fate determined by Rule I
*m*_33_	*m*_33_	Generic	Boolean	true	An entity designed to judge if *m*_18_/*m*_30 _exceeds respective threshold when lin-12 is "wt"/"ko"
*m*_34_	*m*_34_	Generic	Boolean	true	An entity for judging if *m*_18_/*m*_30 _exceeds its threshold when lin-12 is "gf"
*m*_35_	*m*_35_	Generic	Boolean	false	An entity used as a flag to judge if *m*_37 _can get the time epoch when *m*_18 _is over threshold_1
*m*_36_	*m*_36_	Generic	Boolean	false	An entity used as a flag to judge if *m*_38 _can get the time epoch when *m*_18 _is below threshold_1
*m*_37_	Pn.p_fate1_record1	Continuous	Double	0	An entity designed to reserve a time epoch that *m*_18 _exceeds threshold_1
*M*_38_	Pn.p_fate1_record2	Continuous	Double	0	An entity designed to reserve a time epoch that *m*_18 _decreases below threshold_1
*m*_39_	*m*_39_	Generic	Boolean	false	An entity used as a flag to judge if *m*_41 _can get the time epoch when *m*_30 _is over threshold_2
*m*_40_	*m*_40_	Generic	Boolean	false	An entity used as a flag to judge if *m*_42 _can get the time epoch when *m*_30 _is below threshold_2
*m*_41_	Pn.p_fate2_record1	Continuous	Double	0	An entity designed to reserve a time epoch that *m*_30 _exceeds threshold_2
*m*_42_	Pn.p fate2_record2	Continuous	Double	0	An entity designed to reserve a time epoch that *m*_30 _decreases below threshold_2
*m*_43_	Pn.p_m1_interval	Continuous	Double	0	Time difference between *m*_38 _and *m*_37 _that is the longest time interval at the present time epoch
*m*_44_	Pn.p_fate1_init	Continuous	Double	0	Time epoch that *m*_18 _exceeds threshold_1 to the initial one in the time span of *m*_43 _corresponding
*m*_45_	Pn.p_m2_interval	Continuous	Double	0	Time difference between *m*_42 _and *m*_41 _that is the longest time interval at the present time epoch
*m*_46_	Pn.p_fate2_init	Continuous	Double	0	Time epoch that *m*_30 _exceeds threshold_2 corresponding to the initial one in the time span of *m*_45_
-	*lin*3_*init*	Continuous	Double	100	Amount of LIN-3 impulse emanating from AC
-	*Steady_state_time(Gonad)*	Continuous	Double	400	Time epoch of substance that achieve a steady state level
*simultime*	*Simulation_time*	Continuous	Double	2000- getSamplingInterval(simulator)	The length of simulation time

**Table 5:** Properties of processes for the HFPNe model of Figure 6

Process	Process type	Process activity
*p*_25_	Generic	if (IfTime(simulator, steady_state_time(Gonad)) && AC == true) {return true;} else {return false;}

*p*_26_	Generic	if ("gf".equals(lin12)) {return false;} else {return true;}

*p*_27_	Generic	if ("gf".equals(lin12) && (getElapsedTime(simulator) > simultime+getSamplingInterval(simulator))) {return true;} else {return false;}

*p*_28_	Generic	if ((m35 == true && m36 == true) && m18 > = threshold_1) {return true;} else {return false;}

*p*_29_	Generic	if (m18 > = threshold_1 && m35 == false) {return true;} else {return false;}

*p*_30_	Generic	if ((m35 == true && m36 == false && m18 < threshold_1) || (m35 == true && m36 == false && m18 > = threshold_1 && IfTime(simulator, simultime))) {return true;} else {return false;}

*p*_31_	Generic	if (((m39 == true) && (m40 == true)) && (m30 > = threshold_2)) {return true;} else {return false;}

*p*_32_	Generic	if ((m30 > = threshold_2) && (m39 == false)) {return true;} else {return false;}

*p*_33_	Generic	if ((m39 == true && m40 == false && m30 < threshold_2) || (m39 == true && m40 == false && m30 > = threshold_2 && IfTime(simulator, simultime))) {return true;} else {return false;}

*p*_34_	Generic	if ((m35 == true && m36 == true) || (m35 == true && getElapsedTime(simulator)>simultime)) {return true;} else {return false;}

*p*_35_	Generic	if ((m39 == true && m40 == true) || (m39 == true && getElapsedTime(simulator)>simultime)) {return true;} else {return false;}

*p*_36_	Generic	if (getElapsedTime(simulator) > simultime+getSamplingInterval(simulator) && ("gf".equals(lin12) == false)) {return true;} else {return false;}

*p*_37_	Generic	if ((getElapsedTime(simulator) > simultime + getSamplingInterval(simulator)) && "gf".equals(lin12)) {return true;} else {return false;}

Table 5: External Java functions getElapsedTime(simulator), getSamplingInterval(simulator), and IfTime(simulator, time) return the value of elapsed time during simulation, the sampling interval of simulator, and true/false by judging if elapsed time equals to the value of time, respectively. All the functions are written in Pnuts language [48].

**Table 6:** Update functions of connectors for HFPNe model of Figure 6

Connector Name	Connector type	Update function
**Rule I:** if ("gf" .equals(lin12)) == false holds, the connectors are updated as follows:		

*c*_14_(*p*_28_, *m*_35_)	process	return false;

*c*_15_(*p*_28_, *m*_36_)	process	return false;

*c*_16_(*m*_35_, *p*_29_)	process	return true;

*c*_17_(*m*_36_, *p*_30_)	process	return true;

*c*_18_(*p*_29_, *m*_37_)	process	return getElapsedTime(simulator);

*c*_19_(*p*_30_, *m*_38_)	process	return getElapsedTime(simulator);

*c*_20_(*p*_31_, *m*_39_)	process	return false;

*c*_21_(*p*_31_, *m*_40_)	process	return false;

*c*_22_(*m*_39_, *p*_32_)	process	return true;

*c*_23_(*m*_40_, *p*_33_)	process	return true;

*c*_24_(*p*_32_, *m*_41_)	process	return getElapsedTime(simulator);

*c*_25_(*p*_33_, *m*_42_)	process	return getElapsedTime(simulator);

*c*_26_(*p*_34_, *m*_43_)	process	if (m43! = 0 && m43 < m38-m37) {return m38-m37;} else if (m43! = 0 && P3p_m1_interval > = m38-m37) {return m43;} else {return m38-m37;}

*c*_27_(*p*_34_, *m*_44_)	process	if (m44! = 0 && m43 < m38-m37) {return m37;} else if (m44! = 0 && m43 > = m38-m37) {return m44;} else {return m37;}

*c*_28_(*p*_35_, *m*_45_)	process	if (m45! = 0 && m45 < m42-m41) {return m42-m41;} else if (m45! = 0 && m45 > = m42-m41) {return m45;} else {return m42-m41;}

*c*_29_(*p*_35_, *m*_46_)	process	if (m46! = 0 && m45 < m42-m41) {return m41;} else if (m46! = 0 && m45 > = m42-m41) {return m46;} else {return m41;}

*c*_30_(*p*_36_, *m*_32_)	process	if (m43 > = interval && m45 > = interval) {if (m44 < = m46) {return 1;} else {return 2;}} else if (m43 > = interval && m45 < interval) {return 1;} else if (m43 < interval && m45 > = interval) {return 2;} else{return 3;}

*c*_31_(*p*_37_, *m*_32_)	process	if (m43 > = interval && m45 > = interval) {return 1;} else if (m43 > = interval && m45 < interval) {return 1;} else if (m43 < interval && m45 > = interval) {return 2;} else {return 3;}

*c*_32_(*p*_25_, *m*1)	process	lin3_init

**Rule II:** if ("gf" .equals(lin12)) == false holds, the connectors are updated as follows:		
*c*6(*m*_33_, *p*_26_)	process	if (m18 > = threshold_1 || m30 > = threshold_2) {return false;} else {return true;}

*c*7(*m*_18_, *p*_26_)	process	return m18;

*c*8(*m*_30_, *p*_26_)	process	return m30;

*c*9(*p*_26_, *m*_31_)	process	if(m33 == true) {if(m18 > = threshold_1) {return 1;} else if (m30 > = threshold_2) {return 2;} else {return 3;}} else {return m31;}

if ("gf" .equals(lin12)) == true holds, the connectors are updated as follows:		

*c*_10_(*m*_18_, *p*_27_)	process	return m18;

*c*_11_(*m*_34_, *p*_27_)	process	if (m18 > = threshold_1 && m30 > = threshold_2) {return false;} else {return true;}

*c*_12_(*m*_30_, *p*_27_)	process	return m30;

*c*_13_(*p*_27_, *m*_31_)	process	if(m18 < threshold_1 && m30 < threshold_2) {return 3;} else if(m285 > = threshold_1) {return 1;} else {return 2;}

**Figure 8:**
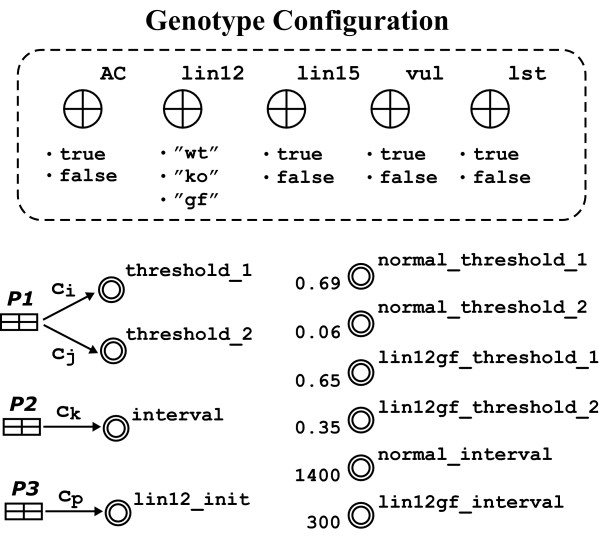
**The panel of Genotype Configuration and the automatic-switching mechanisms to determine the values of *threshold_1*/*threshold_2*, *interval*, and *lin12_init *according to the different genetic conditions**.

Further, several variables are designed to reserve alterable values according to different genotypes (see Table 7). Figure 8 shows a *Genotype Configuration *panel and a structure of auto-switching mechanism. The Genotype Configuration panel includes an AC and four mutant variables. AC, lin15, vul and lst can toggle between true and false. lin12 has three string-type values, i.e., "wt", "ko", "gf", indicating three genetic conditions of wild, knockout and overexpression of *lin-12*.

**Table 7:** Detailed parameters and functions of Figure 8.

Variable name	Update function
*c*_*i*_(*P*_1_, *threshold_*1)	if ("gf".equals(lin12)) {return lin12gf_threshold_1;} else {return normal_threshold_1;}

*c*_*j*_(*P*_1_, *threshold_*2)	if ("gf".equals(lin12)) {return lin12gf_threshold_2;} else {return normal_threshold_2;}

*c*_*k*_(*P*_2_, *interval*)	if ("gf".equals(lin12)) {return lin12gf_interval;} else {return normal_interval;}

*c*_*p*_(*P*_3_, *lin*12_*init*)	if ("gf".equals(lin12)) {return LSMass(2.0,1.0);} else if ("ko".equals(lin12)) {return 0;} else {return LSMass(1.0,1.0);}

Table 7: The auto-switching mechanism of determining the value of *threshold_*1, *threshold_*2, *interval *and expression speed of lin12 under different mutant combinations as shown in the block of "Genotype Configuration" of Figure 8. If lin12 equals to "wt", a generic process *P*_3 _will fire to deposit LSMass(1.0,1.0) as an expression speed of *lin-12*; if lin12 equals to "ko", *P*_3 _will deposit 0; and if lin12 equals to "gf", *P*_3 _will fire to deposit LSMass(2.0,1.0) as the speed denoting an overexpression of *lin-12*. The variables of *threshold_*1, *threshold_*2 and *interval *are designed likewise. All update functions are written in Pnuts language [48]

It is obvious that the combination number is 48 as listed in Table 8. Forty-eight genotypes have the features as follows:

**Table 8:** Summary of the VPC fate patterns of 48 combinations concerning the AC and four mutants.

**RowID**	**AC**	**Genotype**	**Fate Patterns (JA)**	**Fate Patterns (ST)**	**Fate Patterns (STA)**	**Ref**.
			
			*lin-12 lin-15 vul lst *P3.pP4.pP5.pP6.pP7.pP8.p	P3.pP4.pP5.pP6.pP7.pP8.p	P3.pP4.pP5.pP6.pP7.pP8.p	
1^†^	+	wt wt wt wt	[332123]	-	-	[49]

2^†^	+	wt wt wt ko	[331113]	-	-	[35,50]

**[3]**	+	wt wt ko wt	[333333]	[333333]	[331113] [331123] [331133] [332313] [332323] [332333] [333323] [333333]	[32]

4^†^	+	wt wt ko ko	[333333]	-	-	u.d.

**[5]**	+	wt ko wt wt		Unstable pattern (refer to Table 9)		[32]

6^†^	+	wt ko wt ko	[111111]	-	-	[46]

**[7]**	+	wt ko ko wt	[333333]	[333333]	[333333]	[32,37,51]

8	+	wt ko ko ko	[333333]	-	-	n.d.

**[9]**	+	ko wt wt wt	[331113]	[331113]	[311111] [311112] [311113] [321111] [321112] [321113] [331111] [331112] [331113] -	[32]

10^†^	+	ko wt wt ko	[331113]	-	-	u.d.

**[11]**	+	ko wt ko wt	[333333]	[333333]	[311113] [311123] [311133] [331133] [331213] [331223] [332133] [332213] [332223] [333133] [333213] [333223] -	[32]

12	+	ko wt ko ko	[333333]	-	-	n.d.

**[13]**	+	ko ko wt wt	[111111]	[111111]	[111111]	[32]

14	+	ko ko wt ko	[111111]	-	-	n.d.

15	+	ko ko ko wt	[333333]	-	-	n.d.

16	+	ko ko ko ko	[333333]	-	-	n.d.

**[17]**	+	gf wt wt wt	[222122]	[222122]	[122122] [222122] [322122]	[32]

18	+	gf wt wt ko	[221112]	-	-	n.d.

**[19]**	+	gf wt ko wt	[222222]	[222222]	[122222] [222222] [322222]	[32]

20	+	gf wt ko ko	[222222]	-	-	n.d.

**[21]**	+	gf ko wt wt		Unstable pattern (refer to Table 9)		[32]

22	+	gf ko wt ko	[111111]	-	-	n.d.

23	+	gf ko ko wt	[222222]	-	-	n.d.

24	+	gf ko ko ko	[222222]	-	-	n.d.

25^†^	-	wt wt wt wt	[333333]	-	-	[52]

26^†^	-	wt wt wt ko	[333333]	-	-	[50]

27	-	wt wt ko wt	[333333]	-	-	n.d.

28	-	wt wt ko ko	[333333]	-	-	n.d.

**[29]**	-	wt ko wt wt		Unstable pattern (refer to Table 9)		[32]

30	-	wt ko wt ko	[111111]	-	-	n.d.

31	-	wt ko ko wt	[333333]	-	-	n.d.

32	-	wt ko ko ko	[333333]	-	-	n.d.

**[33]**	-	ko wt wt wt	[333333]	[333333]	[333333]	[32]

34	-	ko wt wt ko	[333333]	-	-	n.d.

35	-	ko wt ko wt	[333333]	-	-	n.d.

36	-	ko wt ko ko	[333333]	-	-	n.d.

**[37]**	-	ko ko wt wt	[111111]	[111111]	[111111] [111121] [111131]	[32]

38	-	ko ko wt ko	[111111]	-	-	n.d.

39	-	ko ko ko wt	[333333]	-	-	n.d.

40	-	ko ko ko ko	[333333]	-	-	n.d.

**[41]**	-	gf wt wt wt	[222222]	[222222]	[122222] [222222] [322222]	[32]

42^†^	-	gf wt wt ko	[222222]	-	-	[50]

**[43]**	-	gf wt ko wt	[222222]	[222222]	[222222]	[32,53]

44	-	gf wt ko ko	[222222]	-	-	n.d.

**[45]**	-	gf ko wt wt		Unstable pattern (refer to Table 9)		[32]

46	-	gf ko wt ko	[111111]	-	-	n.d.

47	-	gf ko ko wt	[222222]	-	-	n.d.

48	-	gf ko ko ko	[222222]	-	-	n.d.

Table 8: n.d.: not determined, i.e., the fate pattern is only predicted by the *in silico *model without *in vivo *evidences to support. In *lstko *mutant, all *lst *genes are null: *ark-1*, *lip-1*, *dpy-23*, *lst-1*, *lst-2*, *lst-3*, and *lst-4*. In *vulko *mutant, the genes of *let-23*, *sem-5*, *let-60*, *mpk-1 *are null. u.d.: unpublished data; AC(+/-): anchor cell formed/ablated; 1: 1°; 2: 2°; 3: 3°.

(i) For 15 genotypes whose RowID are indicated by boldface and labeled with square brackets, the fate patterns of each genotype have the *in vivo *experimental data reported in [32] (refer to the column of "**Fate Patterns (ST)**" in Tables 8 and 9).

**Table 9:** Expanded results of four unstable VPC patterns with respect to the results of [9] and [32]

**RowID [5]**			**RowID [21]**		
**Predicted patterns**			**Predicted patterns**		

**Fate Patterns (JA)**	**Fate Patterns (ST)**	**Fate Patterns (STA)**	**Fate Patterns (JA)**	**Fate Patterns (ST)**	**Fate Patterns (STA)**

[1/2,1/2,2,1,2,1/2] (Total fate number: 8)	[1/2,1/2,2,1,2,1/2]	[1/2,1/2,2,1,2,1/2] +*a*	[1/2,1/2,2,1,2,1/2] (Total fate number: 8)	[2,1/2,2,1,2,1/2]	[2,1/2,2,1,2,1/2] +*a*

[112121] [122121] [212121]	[112121] [112122] [122121] [122122] [212121] [212122] [222121] [222122]	[112121] [112122] [112123] [122121] [122122] [212121] [212122] [212123] [222121] [222122] [312121] [312122] [312123]	[112121] [122121] [212121]	[212121] [212122] [222121] [222122]	[212121] [212122] [222121] [222122]

**Total fate number: 3**			**Total fate number: 3**		

**RowID [29]**			**RowID [45]**		

**Predicted patterns**			**Predicted patterns**		

**Fate Patterns (JA)**	**Fate Patterns (ST)**	**Fate Patterns (STA)**	**Fate Patterns (JA)**	**Fate Patterns (ST)**	**Fate Patterns (STA)**

[1/2,1/2,1/2,1/2,1/2,1/2]	[1/2,1/2,1/2,1/2,1/2,1/2]	[1/2,1/2,1/2,1/2,1/2,1/2]	[1/2,1/2,1/2,1/2,1/2,1/2]	[1/2,1/2,1/2,1/2,1/2,1/2]	[1/2,1/2,1/2,1/2,1/2,1/2]

(Total fate number: 64)		+*a*	(Total fate number: 64)		+*a*

[111111] [111112]	[111111] [111112]	[111111] [111112]	[111111] [111112]	[111111] [111112]	[111111] [111112]
[111121] [111211]	[111121] [111122]	[111121] [111122]	[111121] [111211]	[111121] [111122]	[111121] [111122]
[111212] [111221]	[111211] [111212]	[111211] [111212]	[111212] [111221]	[111211] [111212]	[111211] [111212]
[112111] [112112]	[111221] [111222]	[111221] [111222]	[112111] [112112]	[111221] [111222]	[111221] [111222]
[112121] [112211]	[112111] [112112]	[112111] [112112]	[112121] [112211]	[112111] [112112]	[112111] [112112]
[112212] [121111]	[112121] [112122]	[112121] [112122]	[112212] [121111]	[112121] [112122]	[112121] [112122]
[121112] [121121]	[112211] [112212]	[112211] [112212]	[121112] [121121]	[112211] [112212]	[112211] [112212]
[121211] [121212]	[112221] [112222]	[112221] [112222]	[121211] [121212]	[112221] [112222]	[112221] [112222]
[121221] [122111]	[121111] [121112]	[121111] [121112]	[121221] [122111]	[121111] [121112]	[121111] [121112]
[122112] [122121]	[121121] [121122]	[121121] [121122]	[122112] [122121]	[121121] [121122]	[121121] [121122]
[211111] [211112]	[121211] [121212]	[121131] [121211]	[211111] [211112]	[121211] [121212]	[121211] [121212]
[211121] [211211]	[121221] [121222]	[121212] [121221]	[211121] [211211]	[121221] [121222]	[121221] [121222]
[211212] [211221]	[122111] [122112]	[121222] [122111]	[211212] [211221]	[122111] [122112]	[122111] [122112]
[212111] [212112]	[122121] [122122]	[122112] [122121]	[212111] [212112]	[122121] [122122]	[122121] [122122]
[212121] [212211]	[122211] [122212]	[122122] [122211]	[212121] [212211]	[122211] [122212]	[122211] [122212]
[212212]	[122221] [122222]	[122212] [122221]	[212212]	[122221] [122222]	[122221] [122222]
	[211111] [211112]	[122222] [211111]		[211111] [211112]	[211111] [211112]
	[211121] [211122]	[211112] [211121]		[211121] [211122]	[211121] [211122]
	[211211] [211212]	[211122] [211211]		[211211] [211212]	[211211] [211212]
	[211221] [211222]	[211212] [211221]		[211221] [211222]	[211221] [211222]
	[212111] [212112]	[211222] [212111]		[212111] [212112]	[212111] [212112]
	[212121] [212122]	[212112] [212121]		[212121] [212122]	[212121] [212122]
	[212211] [212212]	[212122] [212211]		[212211] [212212]	[212211] [212212]
	[212221] [212222]	[212212] [212221]		[212221] [212222]	[212221] [212222]
	[221111] [221112]	[212222] [221111]		[221111] [221112]	[221111] [221112]
	[221121] [221122]	[221112] [221121]		[221121] [221122]	[221121] [221122]
	[221211] [221212]	[221122] [221211]		[221211] [221212]	[221123] [221211]
	[221221] [221222]	[221212] [221221]		[221221] [221222]	[221212] [221221]
	[222111] [222112]	[221222] [222111]		[222111] [222112]	[221222] [221223]
	[222121] [222122]	[222112] [222121]		[222121] [222122]	[221321] [221322]
	[222211] [222212]	[222122] [222211]		[222211] [222212]	[221323] [222111]
	[222221] [222222]	[222212] [222221]		[222221] [222222]	[222112] [222121]
		[222222]			[222122] [222211]
					[222212] [222221]
					[222222] [222312]

**Total fate number: 31**			**Total fate number: 31**		

(ii) For eight genotypes whose RowID are indicated with the superscript (†), the fate patterns of each genotype have been confirmed by model checking approach in the discrete model in [9], which are also consistent with the biological facts [35,46,49-53].

(iii) For the left 25 genotypes, the patterns of each genotype have only been verified in [9], because these genotypes are usually experimentally intractable. The reason is that, the population of double, triple or quadruple mutants might be technically difficult to be generated [9].

(iv) For four genotypes (i.e., RowID 5, 21, 29, and 45) are called *unstable patterns*, because genetic condition will lead to an unstable fate pattern as shown in Table 9.

In the next subsection, we will execute simulation to investigate the characteristics of predicted fate patterns of 48 genotypes, in particular, four genotypes leading to the unstable fate pattern, and to evaluate two rules employed to the VPC fate model by comparing with three simulation targets (i.e., **JA**, **ST**, and **STA**) as shown in Figure 1(b).

### Simulation-based model checking approach of determining VPC specification by using Cell Illustrator

We first demonstrate how to assign parameters to the VPC fate model. Then, we consider three simulation targets to investigate the properties of fate patterns, and to evaluate two proposed rules. Finally, a large number of simulations are performed with the use of "High-Speed Simulation Module" of Cell Illustrator. The simulation results are given in the form of *fitting score *and *variation frequency *of each pattern of 48 genotypes. The fitting score is a percentage of the total numbers of predicted patterns appeared in each genotype); the variation frequency is the number of each appearing fate pattern in the respective genotype during the simulation experiments.

#### Parameter assignment

The VPC fate model in Figure 5 reflects the interrelation of each substance. All parameters for the reaction rates of processes and the steady state of the substance are tuned manually with repeating simulation until concentration behaviors of proteins correspond to the biological facts. Note that it is hard to decide optimal values of these parameters, since data from biological experiments are very insufficient to determine them.

We thus simplify the kinetic parameters to the same speeds *m*_x_*0.1 with noise for the process *p*_*i*_∈ {*p*_1_, *p*_2_, ⋯, *p*_24_} (see Table 2); the speed of the source process  is assigned to 1.0 denoting the production rate of substance. When constructing the quantitative VPC fate model, threshold values (partly shown in Figure 8 and Table 10) of each reaction are assigned as real number, and the parameters for the steady states of the substances induced by the stimulations from anchor cell and hyp7 are carefully tuned by hand. Lots of trial and error operations have been performed repeatedly until appropriate parameters for simulation are determined. The VPC fate model in Figure 5 is available from [54], which can be executed on Cell Illustrator [24] or Java web start software Cell Illustrator Online 4.0 (CIO) [25]. All the parameters have been saved in the csv format, which are also available online at the same website [54].

**Table 10:** Thresholds used in the HFPNe model of Figure 6.

Connector Name	Connector description	Firing threshold
*c*_1_(*m*_11_, *p*_19_)	Active MAPK-1 represses the shedding process of ligand-receptor complex	4.3+*rand*()/10
*C*_2_(*m*_5_, *p*_22_)	Active LIN-3/LET-23 dimer represses the expression of *lst*	0.5+*rand*()/10
*c*_3_(*m*_27_, *p*_6_)	Expressed LST prevents SEM-5 from becoming an active form	0.09+*rand*()/10
*c*_4_(*m*_27_, *p*_7_)	Expressed LST prevents LET-60 from becoming an active form	0.09+*rand*()/10
*c*_5_(*m*_27_, *p*_8_)	Expressed LST prevents MPK-1 from becoming an active form	0.09+*rand*()/10

#### Three simulation targets for validation

##### [Obtaining fate patterns (JA) by using model checker: MOCHA]

Model checking is powerful technique for automatically verifying the system requirements. The essential idea of model checking is that, with an exhaustive exploring of all reachable states and transitions of a modeled system, system properties (expressed as a formal specification) are examined whether the properties are satisfied or not. A model checker (e.g., MOCHA[55]) accepts the modeled system and the specification as its inputs. The checker then outputs yes if the model satisfies the given specification and generates a *counterexample *otherwise. The counterexample indicates why the model does not satisfy the given specification. By repeating operations of revising the errors examined by the counterexamples, the modeled system can be refined to satisfy enough system specifications [1,2].

Fisher *et al*. have firstly applied the model checking approach for validating biological systems of *C. elegans *with biological experiments [9]. They have constructed a discrete and state-based mechanistic model underlying the inductive and lateral signaling crosstalk by using the language of *reactive modules *[13] and MOCHA. Further, they have confirmed that the model can reproduce reported biological behavior observed in 22 genotypes with MOCHA. However, their method has the following weaknesses that are desired to be solved in this paper:

(1) The details of predicted fate patterns of four unstable patterns (RowID 5, 21, 29, and 45) are not explicitly given. That is, only summarized patterns are given. However, it is important to reveal if all the expansions of predicted (summarized) patterns will appear even though the predicted patterns satisfy the given specification. For example, in the case of *ac-; lin-12gf; lin-15ko *double mutants (RowID 45 in Table 8), the predicted pattern is [1/2, 1/2, 1/2, 1/2, 1/2, 1/2 ] in [9]. It is obvious that the pattern includes totally 2^6 ^= 64 possible fate patterns, but it is still ambiguous if all these 64 patterns will be adopted in *in vivo *experiments.

(2) The distribution and variation of individual behavior of predicted fate patterns are not sufficient to understand the features of the fate patterns. In other words, it is necessary to clarify the occurrence probability (called *variation frequency*) of each predicted pattern, which is considered to facilitate obtaining an overall distribution of the predicted fate patterns, especially, the four unstable patterns.

To improve the first weakness, we introduce a new procedure GENERATENECESSARYFATEPATTERNS by repeating the use of "counterexample" to obtain necessary fate patterns on MOCHA.

«PROCEDURE GENERATENECESSARYFATEPATTERNS»

[******]: A state pattern of six VPC fates; * is one of three alternative fates (1°, 2°, and 3°); i.e., [111111] denotes a fate pattern with 1° for all VPCs. In total, there are 729(= 3^6^) fate patterns.

*RS*: A set to store intermediate fate patterns required for a genotype.

*Verify*: A MOCHA procedure to verify if the predication holds for the model. The input is the *RS*. If there is no counterexample for the *RS*, *ϕ *will be returned. If the counterexample is generated for the *RS*, one of the counterexamples will be returned at random.

The new procedure to derive whole fate patterns for a genotype is as follows: 1. *RS *← {[111111]}

2.   **do ***c *← *Verify*(*RS*)

3.      *RS *← *RS *∪ {*c*}

4.   **while ***c *≠ *ϕ*

5. *c *← *Verify*(*RS*\{[111111]})

6. **if ***c *= *ϕ*

7.    return *RS*\{[111111]}

8. **else**

9.    return *RS*

In the above procedure, step 1 is an initialization of *RS*. Steps 2 – 4 are the main part to obtain the fate patterns by repeating the use of "counterexample". Steps 5 – 9 are designed to check if the initial fate pattern is necessary or not. By executing the procedure, the results of required fate patterns of 48 genotypes are summarized in the fourth column of Table 8, and the first and fourth columns of Table 9. The predicted fate patterns of each genotype derived by using our procedure are collectively called **Fate Patterns (JA) **(**JA **for short). It is clear that the number of predicted fate patterns investigated by our method is far smaller than the one summarized by [9] (see Table 9). The source code used to obtain **JA **with MOCHA can be found at the CSML website [54].

##### [Fate patterns (ST) obtained from *in vivo *data]

Sternberg and Horvitz have summarized the VPC fate patterns of 15 genotypes by the observation of anatomy and the cell lineages in living *C. elegans *using Nomarski differential interference contrast optics (see Table 4 in the original paper [32]). We list the fate patterns of these 15 genotypes (refer to the fifth column in Table 8, and the second and fifth columns in Table 9).

##### [Fate patterns (STA) obtained from *in vivo *data including hybrid lineage observations]

In [32], we have noticed that some biological observations of cell lineages have not received enough attentions. These observations are likely difficult to be determined accurately according to the two criteria defined by Sternberg and Horvitz [32,39]. The reason is that some lineages were not interpretable as 1° or 2° by two criteria, i.e., observed lineages may result in possible inaccuracy in the observations which includes mis-scoring adherence to ventral cuticle owing to deformations in cuticle as animals bend, as well as variations in division axes [32]. In some cases, lineages are hybrid, e.g., [S TT] in which [S] is the production of the characteristics of the 3° fate, and [TT] can be the lineage of either the 1° or 2° fate. Sternberg and Horvitz have suggested that hybrid sublineage is a result of imprecise decision, and the specification is allowed to have a number of outcomes. When a system comes close to a certain threshold, it can be considered that partial functions are involved to express the results in the formation of hybrid lineage [39].

Inspired by the observation of hybrid lineage, it seems reasonable to put those uninterpretable experimental data together with the fate patterns of [32] (i.e., **ST**) as the simulation targets for validation. By investigating the experimental results of the vulval cell lineage given in [32], we explain the cell fate of such an uninterpretable lineage as three plausible fate candidates: 1°, 2°, and 3° fates. Thus, the VPC fate pattern including the uninterpretable lineage is extended to three predicted fate patterns (see **STA **block in Figure 1(b)). For example, in the case of *vulko *(RowID 3 in Table 8), we observed such a cell lineage: [[S S] [S S] [S S] [S TT] [S S] [S S] ], in which [S S] is a cell lineage of 3° and [S TT] is a hybrid lineage and is interpretable by neither two criteria defined in [32] nor the method in [9]. This cell lineage is determined by the fate pattern [333?33]. We thus suppose to interpret this VPC fate pattern to three fate pattern extensions of [333133], [333233], and [333333], i.e., extend "?" (uncertain fate) to the 1°, 2°, and 3° fate. All the possible cell fate patterns including extended fate patterns are shown in the column **Fate Patterns (STA) **of Tables 8 and 9, i.e., **STA **is the combination of **ST **and the pattern extensions.

#### Simulation procedures

Here, we discuss several considerations of simulation environment adjustment in order to validate the VPC fate model and evaluate two rules.

When emulating cell stimulations in *in silico *experiments, it is important to adjust the cell to a constant condition before the stimulation. This condition is usually called a *steady state*. For example, simulation time periods of 400 [pt] and 200 [pt] are reserved representing LIN-3 stimulations from AC and hyp7 respectively in the VPC fate model ([pt] is the virtual time unit of the HFPNe model). This is because the concentration of the receptor LET-23 will be kept at a steady state after 200 [pt]. The time interval of the stimulations between AC and hyp7 is designed to investigate respective variation of fate patterns.

Meanwhile, the following is taken into account to emulate real environment as well as assessing the robustness of the model:

(i) The notion of log-normal distribution is introduced. For each process, log-normal distribution is applied as a system noise. Each standard deviation is given in the third column in Table 2, where *LSMass*(*arg*1, *arg*2) denotes the function of log-normal distribution, *arg*1 denotes normal reaction speed without noise, and *arg*2 is the standard deviation denoting the strength of the noise.

(ii) In the case of *lin-15ko *mutant, a little temporal difference between the stimulations of LIN-3 emanating from hyp7 to each Pn.p is designed to investigate the influence of ectopic stimuli.

(iii) The key thresholds involved in Ras/MAPK and LIN-12/Notch signaling pathways are assigned to the variables, which depend on the function of *rand*() that fluctuates randomly between 0.0 and 1.0 (refer to Table 10). Here, uniform random number generated by Mersenne Twister random number generator is used.

We have tried 100, 1,000 and 10,000-run simulation experiments. For all cases, the results clearly show that **Rule I **can generate high fitting score than **Rule II**. Among them, since 10,000-run simulation can present the most precise behaviors of each fate pattern, in this paper we have concentrated on the 10,000-run simulation results to investigate the fitting score and variation frequency of 48 genotypes on the VPC fate model. The simulation experiments are carried out on the workstation of Intel Xeon X5450 (3.0 GHz) processors with 16 Gbytes of memory. The machine has two CPUs which has four cores per CPU (number of total processor cores is eight). The theoretical computational performance is 96 GFLOPS. All simulations were run on Java 1.6 environment. The VPC fate model will take nearly 100 [sec] for one simulation on Cell Illustrator with a script-based simulation engine. It provides detailed visualization of system behaviors. On the other hand, Cell Illustrator is equipped with a "High-Speed Simulation Module" specialized in producing simulation results only. It increases the simulation speed from 10 to 100 times than the script-based one. Hence, the 10,000 simulations can be conducted on a day on average, and the 48 sets can be executed on 48 days on a single core of a processor. We carried out all simulations within 6 days on our computing environment. We use the same model, the same procedures and also the same parameters to evaluate the fate patterns of all 48 genotypes with the three simulation targets (refer to Tables 8 and 9).

## Results and Discussion

We now exhibit and discuss our simulation results on the fitting score of predicted fate patterns and the variation frequency of each appearing fate pattern, especially focusing on the four unstable patterns.

**Figure 9:**
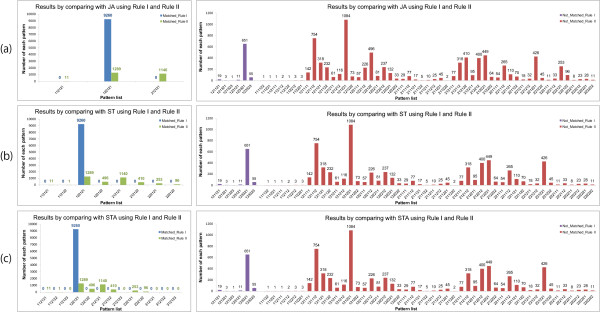
**The simulation results of *lin-15ko *mutants (RowID 5)**.

**Figure 10:**
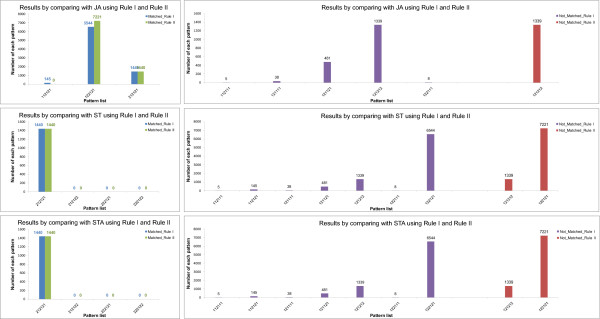
**The simulation results of *lin-12gf; lin-15ko *double mutants (RowID 21)**.

**Figure 11:**
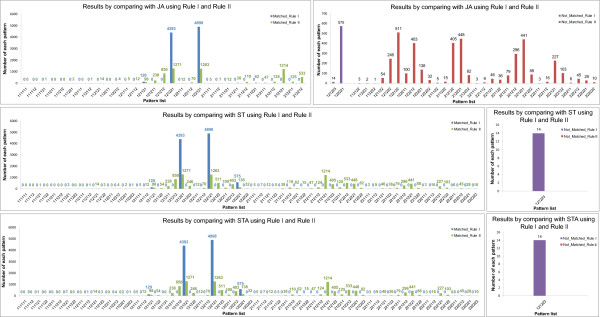
**The simulation results of *ac*- and *lin-15ko *mutant (RowID 29)**.

**Figure 12:**
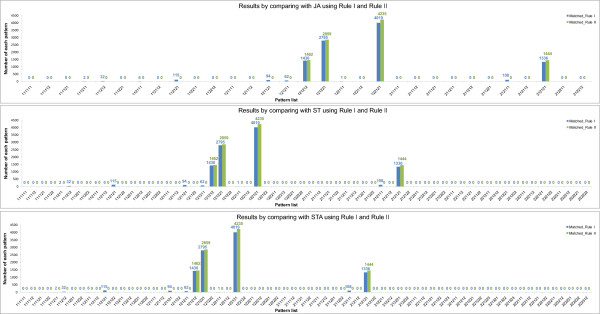
**The simulation results of *ac*- and *lin-12gf; lin-15ko *double mutants (RowID 45)**.

Using the method addressed in the previous sections, we performed the simulation of the VPC fate model. Tables 10, 11, 12, 13, 14 and Additional file 2 exhibit the simulation results of 48 genotypes. Figures 9, 10, 11 and 12 show the graphs of four unstable patterns (RowID 5, 21, 29, and 45), which depict the variation frequency of each predicted pattern and the variation distribution based on simulation results. For example, in Figure 9, the fate pattern [122121] predominantly appeared in the matched results of JA by using **Rule I **and **Rule II**, while the pattern is regarded as a false pattern because it is not observed in ST and STA.

**Table 11:** Details of the simulation results for RowID 5 in Table 9

RuleI_JA				RuleI_ST				RuleI_STA			
Patterns	Num.	Patterns	Num.	Patterns	Num.	Patterns	Num.	Patterns	Num.	Patterns	Num.

[112121]^X^	0			[112121]^X^	0	[212121]^X^	0	[112121]^X^	0	[212123]^X^	0
[122121]^X^	9260			[112122]^X^	0	[212122]^X^	0	[112122]^X^	0	[222121]^X^	0
[212121]^X^	0			[122121]^X^	9260	[222121]^X^	0	[112123]^X^	0	[222122]^X^	0
				[122122]^X^	0	[222122]^X^	0	[122121]^X^	9260	[312121]^X^	0
								[122122]^X^	0	[312122]^X^	0
								[212121]^X^	0	[312123]^X^	0
								[212122]^X^	0		

**Total: 9260 (92.6%)**				**Total: 9260 (92.6%)**				**Total: 9260 (92.6%)**			

[121121]	19			[121121]	19			[121121]	19		
[121321]	3			[121321]	3			[121321]	3		
[121323]	1			[121323]	1			[121323]	1		
[123121]	11			[123121]	11			[123121]	11		
[123321]	651			[123321]	651			[123321]	651		
[123323]	55			[123323]	55			[123323]	55		

**Total: 740 (7.4%)**				**Total: 740 (7.4**%**)**				**Total: 740 (7.4%)**			

RuleII_JA				RuleII_ST				RuleII _STA			

Patterns	Num.	Patterns	Num.	Patterns	Num.	Patterns	Num.	Patterns	Num.	Patterns	Num.

[112121]^X^	11			[112121]^X^	11	[212121]^X^	1140	[112121]^X^	11	[212123]^X^	0
[122121]^X^	1289			[112122]^X^	1	[212122]^X^	410	[112122]^X^	1	[222121]^X^	253
[212121]^X^	1140			[122121]^X^	1289	[222121]^X^	253	[112123]^X^	0	[222122]^X^	96
				[122122]^X^	496	[222122]^X^	96	[122121]^X^	1289	[312121]^X^	0
								[122122]^X^	496	[312122]^X^	0
								[212121]^X^	1140	[312123]^X^	0
								[212122]^X^	410		

**Total: 2440 (24.4%)**				**Total: 3696 (36.96%)**				**Total: 3696 (36.96%)**			

[111122]	1	[211212]	25	[111122]	1	[211222]	2	[111122]	1	[211222]	2
[111221]	1	[211221]	45	[111221]	1	[212111]	77	[111221]	1	[212111]	77
[112111]	1	[211222]	2	[112111]	1	[212112]	318	[112111]	1	[212112]	318
[112112]	2	[212111]	77	[112112]	2	[212211]	95	[112112]	2	[212211]	95
[112122]	1	[212112]	318	[112212]	3	[212212]	400	[112212]	3	[212212]	400
[112212]	3	[212122]	410	[112221]	3	[212221]	449	[112221]	3	[212221]	449
[112221]	3	[212211]	95	[121111]	142	[212222]	64	[121111]	142	[212222]	64
[121111]	142	[212212]	400	[121112]	754	[221111]	54	[121112]	754	[221111]	54
[121112]	754	[212221]	449	[121121]	318	[221112]	265	[121121]	318	[221112]	265
[121121]	318	[212222]	64	[121122]	232	[221121]	110	[121122]	232	[221121]	110
[121122]	232	[221111]	54	[121211]	61	[221122]	70	[121211]	61	[221122]	70
[121211]	61	[221112]	265	[121212]	116	[221211]	18	[121212]	116	[221211]	18
[121212]	116	[221121]	110	[121221]	1084	[221212]	32	[121221]	1084	[221212]	32
[121221]	1084	[221122]	70	[121222]	73	[221221]	426	[121222]	73	[221221]	426
[121222]	73	[221211]	18	[122111]	57	[221222]	45	[122111]	57	[221222]	45
[122111]	57	[221212]	32	[122112]	226	[222111]	11	[122112]	226	[222111]	11
[122112]	226	[221221]	426	[122211]	81	[222112]	33	[122211]	81	[222112]	33
[122122]	496	[221222]	45	[122212]	237	[222211]	8	[122212]	237	[222211]	8
[122211]	81	[222111]	11	[122221]	132	[222212]	23	[122221]	132	[222212]	23
[122212]	237	[222112]	33	[122222]	33	[222221]	28	[122222]	33	[222221]	28
[122221]	132	[222121]	253	[211111]	29	[222222]	11	[211111]	29	[222222]	11
[122222]	33	[222122]	96	[211112]	77			[211112]	77		
[211111]	29	[222211]	8	[211121]	17			[211121]	17		
[211112]	77	[222212]	23	[211122]	5			[211122]	5		
[211121]	17	[222221]	28	[211211]	10			[211211]	10		
[211122]	5	[222222]	11	[211212]	25			[211212]	25		
[211211]	10			[211221]	45			[211221]	45		

**Total: 7560 (75.6%)**				**Total: 6304 (63.4%)**				**Total: 6304 (63.4%)**			

Table 11: X denotes predicted fate pattern, which is also used in Table 12–14.

**Table 12:** Details of the simulation results for RowID 21 in Table 9

RuleI_JA				RuleI_ST				RuleI_STA			
Patterns	Num.	Patterns	Num.	Patterns	Num.	Patterns	Num.	Patterns	Num.	Patterns	Num.

[112121]^X^	145	[212121]^X^	1440	[212121]^X^	1440	[222121]^X^	0	[212121]^X^	1440	[222121]^X^	0
[122121]^X^	6544			[212122]^X^	0	[222122]^X^	0	[212122]^X^	0	[222122]^X^	0

**Total: 8129 (81.29%)**				**Total: 1440 (14.4%)**				**Total: 1440 (14.4%)**			

[112111]	5	[122111]	8	[112111]	5	[121212]	1339	[112111]	5	[121212]	1339
[121111]	38			[112121]	145	[122111]	8	[112121]	145	[122111]	8
[121121]	481			[121111]	38	[122121]	6544	[121111]	38	[122121]	6544
[121212]	1339			[121121]	481			[121121]	481		

**Total: 1871 (18.71%)**				**Total: 8560 (85.6%)**				**Total: 8560 (85.6%)**			

RuleII_JA				RuleII_ST				RuleII_STA			

Patterns	Num.	Patterns	Num.	Patterns	Num.	Patterns	Num.	Patterns	Num.	Patterns	Num.

[112121]^X^	0	[212121]^X^	1440	[212121]^X^	1440	[222121]^X^	0	[212121]^X^	1440	[222121]^X^	0
[122121]^X^	7221			[212122]^X^	0	[222122]^X^	0	[212122]^X^	0	[222122]^X^	0

**Total: 8661 (86.61%)**				**Total: 1440 (14.4%)**				**Total: 1440 (14.4%)**			

[121212]	1339			[121212]	1339	[122121]	7221	[121212]	1339	[122121]	7221

**Total: 1339 (13.39%)**				**Total: 8560 (85.6%)**				**Total: 8560 (85.6%)**			

**Table 13:** Details of the simulation results for RowID 29 in Table 9

RuleI_JA				RuleI_ST				RuleI_STA			
Patterns	Num.	Patterns	Num.	Patterns	Num.	Patterns	Num.	Patterns	Num.	Patterns	Num.

[111111]^X^	0			[111111]^X^	0	[211112]^X^	0	[111111]^X^	0	[211111]^X^	0
[111112]^X^	0			[111112]^X^	0	[211121]^X^	0	[111112]^X^	0	[211112]^X^	0
[111121]^X^	0			[111121]^X^	0	[211122]^X^	0	[111121]^X^	0	[211121]^X^	0
[111211]^X^	0			[111122]^X^	0	[211211]^X^	0	[111122]^X^	0	[211122]^X^	0
[111212]^X^	0			[111211]^X^	0	[211212]^X^	0	[111211]^X^	0	[211211]^X^	0
[111221]^X^	0			[111212]^X^	0	[211221]^X^	0	[111212]^X^	0	[211212]^X^	0
[112111]^X^	0			[111221]^X^	0	[211222]^X^	0	[111221]^X^	0	[211221]^X^	0
[112112]^X^	0			[111222]^X^	0	[212111]^X^	0	[111222]^X^	0	[211222]^X^	0
[112121]^X^	0			[112111]^X^	0	[212112]^X^	0	[112111]^X^	0	[212111]^X^	0
[112211]^X^	0			[112112]^X^	0	[212121]^X^	0	[112112]^X^	0	[212112]^X^	0
[112212]^X^	0			[112121]^X^	0	[212122]^X^	0	[112121]^X^	0	[212121]^X^	0
[121111]^X^	0			[112122]^X^	0	[212211]^X^	0	[112122]^X^	0	[212122]^X^	0
[121112]^X^	0			[112211]^X^	0	[212212]^X^	0	[112211]^X^	0	[212211]^X^	0
[121121]^X^	120			[112212]^X^	0	[212221]^X^	0	[112212]^X^	0	[212212]^X^	0
[121211]^X^	0			[112221]^X^	0	[212222]^X^	0	[112221]^X^	0	[212221]^X^	0
[121212]^X^	0			[112222]^X^	0	[221111]^X^	0	[112222]^X^	0	[212222]^X^	0
[121221]^X^	4393			[121111]^X^	0	[221112]^X^	0	[121111]^X^	0	[221111]^X^	0
[122111]^X^	0			[121112]^X^	0	[221121]^X^	0	[121112]^X^	0	[221112]^X^	0
[122112]^X^	0			[121121]^X^	120	[221122]^X^	0	[121121]^X^	120	[221121]^X^	0
[122121]^X^	4898			[121122]^X^	0	[221211]^X^	0	[121122]^X^	0	[221122]^X^	0
[211111]^X^	0			[121211]^X^	0	[221212]^X^	0	[121131]^X^	0	[221211]^X^	0
[211112]^X^	0			[121212]^X^	0	[221221]^X^	0	[121211]^X^	0	[221212]^X^	0
[211121]^X^	0			[121221]^X^	4393	[221222]^X^	0	[121212]^X^	0	[221221]^X^	0
[211211]^X^	0			[121222]^X^	0	[222111]^X^	0	[121221]^X^	4393	[221222]^X^	0
[211212]^X^	0			[122111]^X^	0	[222112]^X^	0	[121222]^X^	0	[222111]^X^	0
[211221]^X^	0			[122112]^X^	0	[222121]^X^	0	[122111]^X^	0	[222112]^X^	0
[212111]^X^	0			[122121]^X^	4898	[222122]^X^	0	[122112]^X^	0	[222121]^X^	0
[212112]^X^	0			[122122]^X^	0	[222211]^X^	0	[122121]^X^	4898	[222122]^X^	0
[212121]^X^	0			[122211]^X^	0	[222212]^X^	0	[122122]^X^	0	[222211]^X^	0
[212211]^X^	0			[122212]^X^	0	[222221]^X^	0	[122211]^X^	0	[222212]^X^	0
[212212]^X^	0			[122221]^X^	575	[222222]^X^	0	[122212]^X^	0	[222221]^X^	0
				[122222]^X^	0			[122221]^X^	575	[222222]^X^	0
				[211111]^X^	0			[122222]^X^	0		

**Total: 9411 (94.11%)**				**Total: 9986 (99.86%)**				**Total: 9986 (99.86%)**			

[121223]	14	[122221]	575	[121223]	14			[121223]	14		

**Total: 589 (5.89**%**)**				**Total: 14 (0.14**%**)**				**Total: 14 (0.14**%**)**			

RuleII_JA				RuleII_ST				RuleII_STA			

Patterns	Num.	Patterns	Num.	Patterns	Num.	Patterns	Num.	Patterns	Num.	Patterns	Num.

[111111]^X^	0			[111111]^X^	0	[211112]^X^	7	[111111]^X^	0	[211111]^X^	5
[111112]^X^	0			[111112]^X^	0	[211121]^X^	12	[111112]^X^	0	[211112]^X^	7
[111121]^X^	1			[111121]^X^	1	[211122]^X^	5	[111121]^X^	1	[211121]^X^	12
[111211]^X^	0			[111122]^X^	0	[211211]^X^	38	[111122]^X^	0	[211122]^X^	5
[111212]^X^	0			[111211]^X^	0	[211212]^X^	110	[111211]^X^	0	[211211]^X^	38
[111221]^X^	3			[111212]^X^	0	[211221]^X^	62	[111212]^X^	0	[211212]^X^	110
[112111]^X^	0			[111221]^X^	3	[211222]^X^	15	[111221]^X^	3	[211221]^X^	62
[112112]^X^	1			[111222]^X^	0	[212111]^X^	47	[111222]^X^	0	[211222]^X^	15
[112121]^X^	14			[112111]^X^	0	[212112]^X^	124	[112111]^X^	0	[212111]^X^	47
[112211]^X^	0			[112112]^X^	1	[212121]^X^	1214	[112112]^X^	1	[212112]^X^	124
[112212]^X^	4			[112121]^X^	14	[212122]^X^	405	[112121]^X^	14	[212121]^X^	1214
[121111]^X^	5			[112122]^X^	3	[212211]^X^	125	[112122]^X^	3	[212122]^X^	405
[121112]^X^	12			[112211]^X^	0	[212212]^X^	533	[112211]^X^	0	[212211]^X^	125
[121121]^X^	98			[112212]^X^	4	[212221]^X^	448	[112212]^X^	4	[212212]^X^	533
[121211]^X^	238			[112221]^X^	2	[212222]^X^	82	[112221]^X^	2	[212221]^X^	448
[121212]^X^	850			[112222]^X^	1	[221111]^X^	3	[112222]^X^	1	[212222]^X^	82
[121221]^X^	1271			[121111]^X^	5	[221112]^X^	9	[121111]^X^	5	[221111]^X^	3
[122111]^X^	12			[121112]^X^	12	[221121]^X^	46	[121112]^X^	12	[221112]^X^	9
[122112]^X^	70			[121121]^X^	98	[221122]^X^	36	[121121]^X^	98	[221121]^X^	46
[122121]^X^	1263			[121122]^X^	54	[221211]^X^	79	[121122]^X^	54	[221122]^X^	36
[211111]^X^	5			[121211]^X^	238	[221212]^X^	296	[121131]^X^	0	[221211]^X^	79
[211112]^X^	7			[121212]^X^	850	[221221]^X^	441	[121211]^X^	238	[221212]^X^	296
[211121]^X^	12			[121221]^X^	1271	[221222]^X^	88	[121212]^X^	850	[221221]^X^	441
[211211]^X^	38			[121222]^X^	246	[222111]^X^	3	[121221]^X^	1271	[221222]^X^	88
[211212]^X^	110			[122111]^X^	12	[222112]^X^	16	[121222]^X^	246	[222111]^X^	3
[211221]^X^	62			[122112]^X^	70	[222121]^X^	227	[122111]^X^	12	[222112]^X^	16
[212111]^X^	47			[122121]^X^	1263	[222122]^X^	103	[122112]^X^	70	[222121]^X^	227
[212112]^X^	124			[122122]^X^	511	[222211]^X^	6	[122121]^X^	1263	[222122]^X^	103
[212121]^X^	1214			[122211]^X^	100	[222212]^X^	45	[122122]^X^	511	[222211]^X^	6
[212211]^X^	125			[122212]^X^	403	[222221]^X^	28	[122211]^X^	100	[222212]^X^	45
[212212]^X^	533			[122221]^X^	138	[222222]^X^	10	[122212]^X^	403	[222221]^X^	28
				[122222]^X^	32			[122221]^X^	138	[222222]^X^	10
				[211111]^X^	5			[122222]^X^	32		

**Total: 6119 (61.19**%**)**				**Total: 10000 (100**%**)**				**Total: 10000 (100**%**)**			

[112122]	3	[122211]	100								
[112221]	2	[122212]	403	[212122]	405	[221121]	46	[221222]	88	[222211]	6
[112222]	1	[122221]	138	[212221]	448	[221122]	36	[222111]	3	[222212]	45
[121122]	54	[122222]	32	[212222]	82	[221211]	79	[222112]	16	[222221]	28
[121222]	246	[211122]	5	[221111]	3	[221212]	296	[222121]	227	[222222]	10
[122122]	511	[211222]	15	[221112]	9	[221221]	441	[222122]	103		

								**Total: 3881 (38.81%)**			

**Table 14:** Details of the simulation results for RowID 45 in Table 9

RuleI_JA				RuleI_ST				RuleI_STA			
Patterns	Num.	Patterns	Num.	Patterns	Num.	Patterns	Num.	Patterns	Num.	Patterns	Num.

[111111]^X^	0			[111111]^X^	0	[211122]^X^	0	[111111]^X^	0	[211122]^X^	0
[111112]^X^	0			[111112]^X^	0	[211211]^X^	0	[111112]^X^	0	[211211]^X^	0
[111121]^X^	0			[111121]^X^	0	[211212]^X^	0	[111121]^X^	0	[211212]^X^	0
[111211]^X^	2			[111122]^X^	0	[211221]^X^	0	[111122]^X^	0	[211221]^X^	0
[111212]^X^	32			[111211]^X^	2	[211222]^X^	0	[111211]^X^	2	[211222]^X^	0
[111221]^X^	0			[111212]^X^	32	[212111]^X^	108	[111212]^X^	32	[212111]^X^	108
[112111]^X^	6			[111221]^X^	0	[212112]^X^	0	[111221]^X^	0	[212112]^X^	0
[112112]^X^	0			[111222]^X^	0	[212121]^X^	1336	[111222]^X^	0	[212121]^X^	1336
[112121]^X^	115			[112111]^X^	6	[212122]^X^	0	[112111]^X^	6	[212122]^X^	0
[112211]^X^	0			[112112]^X^	0	[212211]^X^	0	[112112]^X^	0	[212211]^X^	0
[112212]^X^	0			[112121]^X^	115	[212212]^X^	0	[112121]^X^	115	[212212]^X^	0
[121111]^X^	0			[112122]^X^	0	[212221]^X^	0	[112122]^X^	0	[212221]^X^	0
[121112]^X^	0			[112211]^X^	0	[212222]^X^	0	[112211]^X^	0	[212222]^X^	0
[121121]^X^	94			[112212]^X^	0	[221111]^X^	0	[112212]^X^	0	[221111]^X^	0
[121211]^X^	62			[112221]^X^	0	[221112]^X^	0	[112221]^X^	0	[221112]^X^	0
[121212]^X^	1430			[112222]^X^	0	[221121]^X^	0	[112222]^X^	0	[221121]^X^	0
[121221]^X^	2795			[121111]^X^	0	[221122]^X^	0	[121111]^X^	0	[221122]^X^	0
[122111]^X^	1			[121112]^X^	0	[221211]^X^	0	[121112]^X^	0	[221123]^X^	0
[122112]^X^	0			[121121]^X^	94	[221212]^X^	0	[121121]^X^	94	[221211]^X^	0
[122121]^X^	4019			[121122]^X^	0	[221221]^X^	0	[121122]^X^	0	[221212]^X^	0
[211111]^X^	0			[121211]^X^	62	[221222]^X^	0	[121211]^X^	62	[221221]^X^	0
[211112]^X^	0			[121212]^X^	1430	[222111]^X^	0	[121212]^X^	1430	[221222]^X^	0
[211121]^X^	0			[121221]^X^	2795	[222112]^X^	0	[121221]^X^	2795	[221223]^X^	0
[211211]^X^	0			[121222]^X^	0	[222121]^X^	0	[121222]^X^	0	[221321]^X^	0
[211212]^X^	0			[122111]^X^	1	[222122]^X^	0	[122111]^X^	1	[221322]^X^	0
[211221]^X^	0			[122112]^X^	0	[222211]^X^	0	[122112]^X^	0	[221323]^X^	0
[212111]^X^	108			[122121]^X^	4019	[222212]^X^	0	[122121]^X^	4019	[222111]^X^	0
[212112]^X^	0			[122122]^X^	0	[222221]^X^	0	[122122]^X^	0	[222112]^X^	0
[212121]^X^	1336			[122211]^X^	0	[222222]^X^	0	[122211]^X^	0	[222121]^X^	0
[212211]^X^	0			[122212]^X^	0			[122212]^X^	0	[222122]^X^	0
[212212]^X^	0			[122221]^X^	0			[122221]^X^	0	[222211]^X^	0
				[122222]^X^	0			[122222]^X^	0	[222212]^X^	0
				[211111]^X^	0			[211111]^X^	0	[222221]^X^	0
				[211112]^X^	0			[211112]^X^	0	[222222]^X^	0
				[211121]^X^	0			[211121]^X^	0	[222312]^X^	0

**Total: 10000 (100**%**)**				**Total: 10000 (100**%**)**				**Total: 10000 (100**%**)**			

RuleII_JA				RuleII_ST				RuleII_STA			

Patterns	Num.	Patterns	Num.	Patterns	Num.	Patterns	Num.	Patterns	Num.	Patterns	Num.

[111111]^X^	0			[111111]^X^	0	[211122]^X^	0	[111111]^X^	0	[211122]^X^	0
[111112]^X^	0			[111112]^X^	0	[211211]^X^	0	[111112]^X^	0	[211211]^X^	0
[111121]^X^	0			[111121]^X^	0	[211212]^X^	0	[111121]^X^	0	[211212]^X^	0
[111211]^X^	0			[111122]^X^	0	[211221]^X^	0	[111122]^X^	0	[211221]^X^	0
[111212]^X^	0			[111211]^X^	0	[211222]^X^	0	[111211]^X^	0	[211222]^X^	0
[111221]^X^	0			[111212]^X^	0	[212111]^X^	0	[111212]^X^	0	[212111]^X^	0
[112111]^X^	0			[111221]^X^	0	[212112]^X^	0	[111221]^X^	0	[212112]^X^	0
[112112]^X^	0			[111222]^X^	0	[212121]^X^	1444	[111222]^X^	0	[212121]^X^	1444
[112121]^X^	0			[112111]^X^	0	[212122]^X^	0	[112111]^X^	0	[212122]^X^	0
[112211]^X^	0			[112112]^X^	0	[212211]^X^	0	[112112]^X^	0	[212211]^X^	0
[112212]^X^	0			[112121]^X^	0	[212212]^X^	0	[112121]^X^	0	[212212]^X^	0
[121111]^X^	0			[112122]^X^	0	[212221]^X^	0	[112122]^X^	0	[212221]^X^	0
[121112]^X^	0			[112211]^X^	0	[212222]^X^	0	[112211]^X^	0	[212222]^X^	0
[121121]^X^	0			[112212]^X^	0	[221111]^X^	0	[112212]^X^	0	[221111]^X^	0
[121211]^X^	0			[112221]^X^	0	[221112]^X^	0	[112221]^X^	0	[221112]^X^	0
[121212]^X^	1462			[112222]^X^	0	[221121]^X^	0	[112222]^X^	0	[221121]^X^	0
[121221]^X^	2859			[121111]^X^	0	[221122]^X^	0	[121111]^X^	0	[221122]^X^	0
[122111]^X^	0			[121112]^X^	0	[221211]^X^	0	[121112]^X^	0	[221123]^X^	0
[122112]^X^	0			[121121]^X^	0	[221212]^X^	0	[121121]^X^	0	[221211]^X^	0
[122121]^X^	4235			[121122]^X^	0	[221221]^X^	0	[121122]^X^	0	[221212]^X^	0
[211111]^X^	0			[121211]^X^	0	[221222]^X^	0	[121211]^X^	0	[221221]^X^	0
[211112]^X^	0			[121212]^X^	1462	[222111]^X^	0	[121212]^X^	1462	[221222]^X^	0
[211121]^X^	0			[121221]^X^	2859	[222112]^X^	0	[121221]^X^	2859	[221223]^X^	0
[211211]^X^	0			[121222]^X^	0	[222121]^X^	0	[121222]^X^	0	[221321]^X^	0
[211212]^X^	0			[122111]^X^	0	[222122]^X^	0	[122111]^X^	0	[221322]^X^	0
[211221]^X^	0			[122112]^X^	0	[222211]^X^	0	[122112]^X^	0	[221323]^X^	0
[212111]^X^	0			[122121]^X^	4235	[222212]^X^	0	[122121]^X^	4235	[222111]^X^	0
[212112]^X^	0			[122122]^X^	0	[222221]^X^	0	[122122]^X^	0	[222112]^X^	0
[212121]^X^	1444			[122211]^X^	0	[222222]^X^	0	[122211]^X^	0	[222121]^X^	0
[212211]^X^	0			[122212]^X^	0			[122212]^X^	0	[222122]^X^	0
[212212]^X^	0			[122221]^X^	0			[122221]^X^	0	[222211]^X^	0
				[122222]^X^	0			[122222]^X^	0	[222212]^X^	0
				[211111]^X^	0			[211111]^X^	0	[222221]^X^	0
				[211112]^X^	0			[211112]^X^	0	[222222]^X^	0
				[211121]^X^	0			[211121]^X^	0	[222312]^X^	0

**Total: 10000 (100**%**)**				**Total: 10000 (100**%**)**				**Total: 10000 (100**%**)**			

From the variation distribution as shown in Figure 9, [122121] is the fate pattern that can be most easily observed in *in vivo *experiments because of the high appearance. In contrast, [112121] is likely difficult to be monitored due to the relative low variation frequency. Each graph comprises three parts of comparison results obtained from JA, ST and STA (see (a), (b), and (c) in Figure 9) by using both **Rule I **and **Rule II**. In each part, the variation frequency results of matched patterns are given on the left side while that of unmatched patterns are exhibited on the right side. Figures 10, 11 and 12 are built up in the same way.

From the simulation results, we draw the following conclusions:

(1) **Rule I **gives more genotypes (RowID) with high fitting scores nearly 100% than **Rule II **to cover predicted patterns of three simulation targets. In more details,

(i) For 44 genotypes (i.e., the genotypes without unstable fate patterns), each genotype can generate the fitting score nearly 95%, in which the fitting scores of 41 genotypes are all 100% (refer to Additional File 1) by using both **Rule I **and **Rule II**. In other words, our model shows good robustness, and can produce stable cell fate patterns even we intentionally add the noise to capture the behaviors of the model fluctuations.

(ii) For the left three genotypes of four unstable fate patterns, *lin-15ko *mutant (RowID 5) produces all 92.6% for JA, ST and STA by using **Rule I**, as opposed to 24.4% (for JA) and 36.96% (for ST and STA) by using **Rule II**; *ac-; lin-15ko *mutant (RowID 29) produces high coverage of 94.11% (for JA) and 99.86% (for both ST and STA); and *ac-; lin-12gf; lin-15ko *double mutants (RowID 45) generates all 100% with both rules for three simulation targets.

(iii) For *lin-15; lin-12ko *double mutants (RowID 21), the fitting scores in the results of ST and STA drop to a quite low value below 15% by using either **Rule I **or **Rule II**. This is because P3.p is allowed to adopt the 1° fate in JA (*in silico *model), but is not allowed in ST and STA (*in vivo *model). This fact has also been confirmed in the preceding study [9] that *in silico *model can faithfully produce the 1° fate for P3.p. From these observations, it can be considered that P3.p has a greater possibility to adopt the 1° fate, if the number of animals for the *in vivo *experiments can be increased to 10,000. In this way, the fitting score can be exactly increased to exceed more than 81% from the present 15%.

(2) For *lin-15ko *(RowID 5) and *ac-;lin-15ko *(RowID 29) genotypes, we can see that nearly 10% patterns in JA, ST and STA are not matched (refer to Tables 11 and 13). These patterns still have the possibility to be examined in *in vivo *experiments by means of enlarging animal population.

(3) Since the experiment data about hybrid lineages has not been taken into account as we have pointed out, we interpret these uninterpretable lineages to three fate pattern extensions as listed in the column of STA which includes more extensive possible fate patterns than ST. In the simulation results, the results of *lin-12ko *mutant (RowID 9) interest us (refer to the Additional file 1). We can find that the fate pattern [331133] appeared in our simulation results of STA, which is the extended fate pattern from the hybrid lineage data exhibited in [32]. This hybrid lineage gives new insights on the imprecise fate decision and the fate specification mechanisms when a system comes close to a certain threshold.

Taking these observations together, we conclude that **Rule I **relying on the temporal interval can be considered more reasonable and proper in evaluating the VPC fate specification than **Rule II**.

## Conclusion

The contribution of this paper is a novel method of modeling and simulating biological systems with the use of model checking approach on the hybrid functional Petri net with extension. A quantitative HFPNe model for the vulval development is constructed based on the literature. Then we employ two major biological fate determination rules to the quantitative model. These two rules are investigated by applying model checking approach in a quantitative manner. Three simulation targets of this model are considered: The first one is the fate patterns obtained by improving the qualitative method of Fisher *et al*. [9]; the second target is the fate patterns summarized by Sternberg and Horvitz [32]; and the last one is derived from the biological experiments in [32] including the hybrid lineage data. We have performed 480,000 simulations on the quantitative HFPNe model by using Cell Illustrator. We have examined the consistency and the correctness of the model, and evaluated the two rules of VPC fate specification. We consider that this computational experiment and the biological evaluation could not be easily put into practice without the HFPNe modeling method and the functions of Cell Illustrator, especially, the "High-Speed Simulation Module". Finally, *in silico *simulation results have been given in the form of the fitting score and the variation frequency of each pattern. We have discussed the results and summarized several plausible explanations.

## Appendix: Abbreviations

AC: (gonadal anchor cell); ac-: (absence of an anchor cell); EGFR: (the epidermal growth factor receptor); HFPNe: (hybrid functional Petri net with extension); JA: (fate patterns obtained with our extended method from the discrete model of Fisher *et al*. [9]); LBS: (LAG-1 binding site); lst: (lateral signal target); MAPK: (mitogen-activated protein kinases); ST: (fate patterns summarized by Sternberg and Horvitz [32]); STA: (fate pattern combinations consisting of ST and the pattern extensions); VPC:(vulval precursor cell); synMuv: (synthetic Multivulva);

## Authors' contributions

The basic idea was considered by MN and further developed by CL and MN. CL created biological diagram of multiple signaling pathways of *C. elegans *depicting VPC fate specification in Figure 4. CL and KU constructed the original HFPNe model in Figures 5 and 6. The whole HFPNe model was refined by CL and MN. CL and MN evaluated the results of simulation and validated the performance of HFPNe model. SM supervised the whole study. The final manuscript was read and approved by all authors.

## Supplementary Material

Additional File 1 **Preliminary notations and mathematical definitions of Rule I and Rule II**. The data provide the details of preliminary notations and mathematical definitions of two rules to determine the cell fate. Click here for file

Additional File 2 **Simulation results of 44 genotypes**. The data give the 10,000 simulation results without unstable fate patterns. Yellowish region means that corresponding genotype possesses experimental evidences exhibited in [32]. Bright yellow cells give the fitting scores of predicted fate patterns. Click here for file
