# Supplementary material for: Simulation-based model checking approach to cell fate specification during Caenorhabditis elegans vulval development by hybrid functional Petri net with extension
Source: BMC Syst Biol. 2009 Apr 27;3:42. doi: 10.1186/1752-0509-3-42 (PMC2691733; doi:10.1186/1752-0509-3-42)
Supplement: Additional File 1 — Preliminary notations and mathematical definitions of Rule I and Rule II. The data provide the details of preliminary notations and mathematical definitions of two rules to determine the cell fate. [file 1752-0509-3-42-S1.pdf]

Preliminary Notations:

(1) Let PN_e denote the whole VPC fate model shown in Figure 5. Let $PN_{e_{sg}}$ be a single VPC fate model shown in Figure 6 whose six copies are embedded in PN_e.

(2) Let *threshold_1* be the criterion of PN_e for determining the 1° fate. Let *threshold_2* be the criterion for determining the 2° fate.

(3) The *interval* is a variable used for the comparison with *interval_1* and *interval_2*.

(4) A time course from the time epoch τ_i to $\tau_{i+interval}$ is denoted as $[\tau_i, \tau_{i+interval})$. τ_i is the first time epoch inducing such an over-threshold state whose length *interval_1* is longer than or equal to the given *interval*; τ_j is the first time epoch inducing such an over-threshold state whose length *interval_2* is longer than or equal to the given *interval*.

(5) Let *simulation_time* be a given simulation time period. □

Rule I is a biological fate determination rule to determine the final VPC fate of $PN_{e_{sg}}$, where

In the case of *lin-12wt* or *lin-12ko* existing:

Cell fate=

1° : $((m_{18}^{[\tau_i,\tau_{i+interval\_1})} \geq threshold\_1) \wedge (m_{30}^{[\tau_j,\tau_{j+interval\_2})} \geq threshold\_2) \wedge (m_{18}^{\tau_i} \leq m_{30}^{\tau_j}) \wedge (interval\_1 \geq interval) \wedge (interval\_2 \geq interval)) \vee ((m_{18}^{[\tau_i,\tau_{i+interval\_1})} \geq threshold\_1) \wedge (m_{30}^{[\tau_j,\tau_{j+interval})} \leq threshold\_2) \wedge (interval\_1 \geq interval))$

(if both the concentrations of 1° (m_{18}) and 2° (m_{30}) are not less than the threshold values (modeled to two entities: *threshold_1* and *threshold_2*), and the over-threshold states are maintained longer than or equal to a given time length (modeled as *interval*), the corresponding cell fate will be adopted according to the time epoch that the threshold is firstly exceeded; or the concentration of m_{30} has ever decreased below *threshold_2* during $[\tau_j, \tau_{j+interval})$ while m_{18} is kept over *threshold_1* at all times during $[\tau_i, \tau_{i+interval\_1})$, then the 1° fate will be adopted.)

2° : $((m_{18}^{[\tau_i,\tau_{i+interval\_1})} \geq threshold\_1) \wedge (m_{30}^{[\tau_j,\tau_{j+interval\_2})} \geq threshold\_2) \wedge (m_{18}^{\tau_i} > m_{30}^{\tau_j}) \wedge (interval\_1 \geq interval) \wedge (interval\_2 \geq interval)) \vee ((m_{18}^{[\tau_i,\tau_{i+interval})} \leq threshold\_1) \wedge (m_{30}^{[\tau_j,\tau_{j+interval\_2})} \geq threshold\_2) \wedge (interval\_2 \geq interval))$

(if m_{18} and m_{30} exceed *threshold_1* and *threshold_2*, respectively, each over-threshold state is maintained longer than or equal to the given *interval*, and the time epoch that m_{30} exceeds *threshold_2* is earlier than the one of m_{18}; or the concentration of m_{18} has ever decreased below *threshold_1* during $[\tau_i, \tau_{i+interval})$ while m_{30} is kept in an over-threshold state during $[\tau_j, \tau_{j+interval\_2})$, then the 2° fate will be adopted.)

3° : otherwise;

In the case of *lin-12gf* existing:

$$\text{Cell fate}=\begin{cases} 1^\circ: & ((m_{18}^{[\tau_i,\tau_{i+interval\_1})}\geq threshold\_1)\wedge(m_{30}^{[\tau_j,\tau_{j+interval\_2})}\geq threshold\_2)\wedge \\ & (interval\_1\geq interval)\wedge(interval\_2\geq interval))\vee \\ & ((m_{18}^{[\tau_i,\tau_{i+interval\_1})}\geq threshold\_1)\wedge(m_{30}^{[\tau_j,\tau_{j+interval})}\leq threshold\_2)\wedge \\ & (interval\_1\geq interval)) \\ & \text{(if } m_{18} \text{ is greater than or equal to } \textit{threshold\_1}\text{, and the} \\ & \text{over-threshold state is maintained longer than or equal to} \\ & \textit{interval} \text{ without respect to the time course expression of } 2^\circ, \\ & \text{the } 1^\circ \text{ fate will be adopted.)} \\ 2^\circ: & (m_{18}^{[\tau_i,\tau_{i+interval})}\leq threshold\_1)\wedge(m_{30}^{[\tau_j,\tau_{j+interval\_2})}\geq threshold\_2)\wedge \\ & (interval\_2\geq interval) \\ & \text{(if } m_{30} \text{ exceed } \textit{threshold\_2} \text{ and is kept in an over-threshold state all the times} \\ & \text{during } [\tau_j,\tau_{j+interval})\text{, and once the concentration of } 1^\circ \text{ is} \\ & \text{less than } \textit{threshold\_1} \text{ during } [\tau_i,\tau_{i+interval})\text{, the } 2^\circ \text{ fate will be adopted.)} \\ 3^\circ: & \text{otherwise;} \end{cases}$$

□

Rule II is another biological fate determination rule to determine the final VPC fate of $PN_{e_{sg}}$, where

In the case of *lin-12wt* or *lin-12ko* mutant existing:

$$\text{Cell fate}=\begin{cases} 1^\circ: & ((m_{18}^{\tau_i}\geq threshold\_1)\wedge(m_{30}^{\tau_j}\geq threshold\_2)\wedge(m_{18}^{\tau_i}\leq m_{30}^{\tau_j})) \\ & \vee((m_{18}^{\tau_i}\geq threshold\_1)\wedge(m_{30}^{[\tau_j,simulation\_time)}<threshold\_2)) \\ & \text{(Once } m_{18} \text{ firstly exceeds } \textit{threshold\_1}\text{, the } 1^\circ \text{ fate will be} \\ & \text{adopted no matter how } m_{30} \text{ changes.)} \\ 2^\circ: & ((m_{18}^{\tau_i}\geq threshold\_1)\wedge(m_{30}^{\tau_j}\geq threshold\_2)\wedge(m_{18}^{\tau_i}>m_{30}^{\tau_j})) \\ & \vee((m_{18}^{[\tau_j,simulation\_time)}<threshold\_1)\wedge(m_{30}^{\tau_j}\geq threshold\_2)) \\ & \text{(Once } m_{30} \text{ firstly exceeds } \textit{threshold\_2}\text{, the } 2^\circ \text{ fate will be} \\ & \text{adopted no matter how } m_{18} \text{ changes.)} \\ 3^\circ: & \text{otherwise;} \end{cases}$$

In the case of *lin-12gf* existing:

$$\text{Cell fate}=\begin{cases} 1^\circ: & m_{18}^{\tau_i}\geq threshold\_1 \\ & \text{(Once } m_{18} \text{ exceeds } \textit{threshold\_1}\text{, the } 1^\circ \text{ fate will be adopted.)} \\ 2^\circ: & m_{18}^{[\tau_i,simulation\_time)}<threshold\_1 \\ & \text{(if } m_{18} \text{ keeps a low concentration less than } \textit{threshold\_1} \text{ until the end of} \\ & \textit{simulation\_time}\text{, the } 2^\circ \text{ fate will be adopted.)} \\ 3^\circ: & \text{otherwise;} \end{cases}$$

□
